# Termination of the integrated stress response

**DOI:** 10.1126/science.adw5137

**Published:** 2025-11-13

**Authors:** Claudia De Miguel, Sigurdur R. Thorkelsson, Agnieszka Fatalska, George Hodgson, Chao Wang, Anne Bertolotti

**Affiliations:** 1 MRC Laboratory of Molecular Biology, Francis Crick Avenue, Cambridge, CB2 0QH, United Kingdom

## Abstract

Stress responses enable cells to detect, adapt to, and survive challenges. The benefit of these signalling pathways depends on their reversibility. The integrated stress response (ISR) is elicited by phosphorylation of translation initiation factor eIF2, which traps and inhibits rate-limiting translation factor eIF2B thereby attenuating translation initiation. Termination of this pathway thus requires relieving eIF2B from P-eIF2 inhibition. Here, we found that eIF2 phosphatase subunits PPP1R15A and PPP1R15B (R15B) bound P-eIF2 in complex with eIF2B. Biochemical investigations guided by cryo-EM structures of native eIF2-eIF2B and P-eIF2-eIF2B complexes bound to R15B demonstrated that R15B enabled dephosphorylation of otherwise dephosphorylation-incompetent P-eIF2 on eIF2B. This sheds light on ISR termination, revealing that R15B rescues eIF2B from P-eIF2 inhibition, thereby safeguarding translation and cell fitness.

Attenuation of protein synthesis to maintain homeostasis is a rapid and evolutionary conserved response to changes in the cellular environment, essential for survival. It mostly occurs at the level of translation initiation, upon phosphorylation of serine 52 (S52) of the alpha subunit of trimeric eukaryotic translation initiation factor 2 (eIF2α) (1, 2). Phosphorylation of eIF2 is central to cellular fitness and has emerged as a therapeutic target for a broad range of diseases, from cancer to neurodegeneration (3, 4). This essential regulation, also known as the integrated stress response (ISR) (5), has been studied for decades (6), yet how phospho-eIF2 signalling is terminated has remained unclear. This is an important fundamental question because persistent stress signalling is lethal (7, 8).

eIF2 is a trimeric (α, β, γ) GTPase that, when bound to GTP, recruits initiator Met-tRNA to the translation preinitiation complex and leaves after GTP hydrolysis (1, 2). For a new round of translation, eIF2-GDP needs to be recycled into eIF2-GTP by its guanine nucleotide exchange factor (GEF) eIF2B. P-eIF2 binds to eIF2B, sequestering it in an inhibited state (9). In human cells, there is a ~ 30-fold molar excess of eIF2 relative to eIF2B (10). Thus, only minute amounts of eIF2 phosphorylation are required to inhibit eIF2B and protein synthesis. This molecular principle establishes the power of this signalling event.

eIF2B is a decamer composed of two copies of 5 subunits, eIF2B α, β, γ, δ and ε (9). eIF2B α, β and δ form the regulatory core, flanked by eIF2Bγ and the catalytic subunit eIF2Bε on each side (11). Recent structures of human recombinant eIF2-eIF2B and P-eIF2-eIF2B complexes have revealed the mechanism of P-eIF2 inhibition: the substrate eIF2 and the inhibitor P-eIF2 bind to two different sites on eIF2B, defining two structurally different complexes, one catalytic and one inhibited (12, 13).

The phosphatases of eIF2 have been identified through genetic screens in mammalian cells (14, 15). They are composed of the PP1 catalytic subunit, bound to one of two non-catalytic subunits, PPP1R15A (R15A) or PPP1R15B (R15B) (14, 15). Biochemical reconstitutions of eIF2α holophosphatases indicate that R15A and R15B are substrate receptors, conferring selectivity to the holoenzyme (16–18). Most studies of eIF2α phosphatases have been conducted with the N-terminal fragment of eIF2α (16, 19). Recently, we reconstituted an R15B-eIF2 complex with recombinant proteins, revealing that R15B binds to eIF2 at the far end of the complex relative to S52 of eIF2α (18). Studies with R15A yielded similar results (20). There are no structures of R15A or R15B bound to their full substrate.

Free P-eIF2α can be dephosphorylated in vitro by recombinant eIF2 phosphatases (16, 20). However, in cells, eIF2 and P-eIF2 are not always free but bound to other translation factors (1, 2). The structures of human P-eIF2 bound to eIF2B indicate that P-S52 of eIF2α is engaged in intramolecular electrostatic interactions (12, 13), most likely inaccessible to dephosphorylation on eIF2B. This raised an important question: How is eIF2B relieved from this inhibition by P-eIF2 for translation to resume?

## R15B bound eIF2 in complex with eIF2B in human cells

**Fig. 1:**
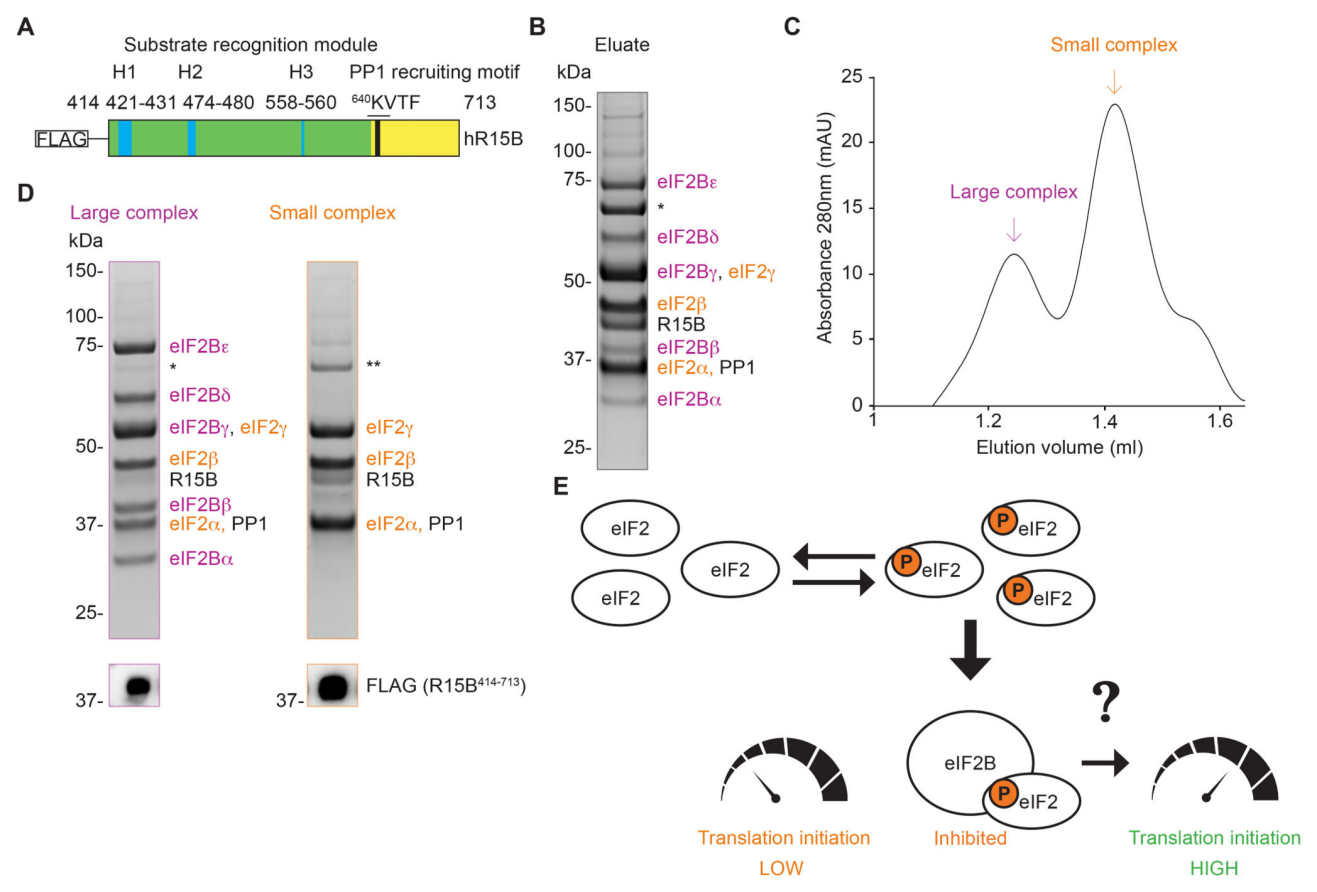
R15B binds to an eIF2B-eIF2 complex. (**A**) Cartoon illustrating the R15B^414-713^ fragment used for purification. H1, H2 and H3 are helical elements important for eIF2 recruitment (18); KVTF: PP1-recruiting motif. (**B**) Coomassie stained gel of an eluate of immunopurified FLAG-R15B^414-713^ expressed in Expi293 cells. Indicated proteins were identified by mass spectrometry (table S1). * Hsp70 1A. (**C**) Representative size exclusion chromatography (SEC) of FLAG-R15B^414-713^ eluate (B). (**D**) Coomassie-stained gel of large and small complex peak fractions from SEC (C). Indicated proteins were identified by mass spectrometry (table S2). * Hsp70 1A. ** ANM5. Immunoblots with FLAG antibody (bottom). (**E**) Cartoon depicting the control of translation initiation by P-eIF2. P-eIF2 binds and sequesters eIF2B in an inhibited state. Because eIF2B is rate-limiting, it is not the abundance of free P-eIF2 but the unavailability of eIF2B that controls translation inhibition. Thus, translation recovery requires the rescue of eIF2B from P-eIF2 inhibition. How this occurs is unknown.

Knowing that the N-terminal 413 residues of R15B are dispensable for eIF2 recruitment and dephosphorylation (17, 18), we expressed FLAG-tagged R15B^414-713^ in human cells and immunopurified native complexes for structural studies (Fig. 1A). R15B co-purified with several proteins, which we identified by mass spectrometry (Fig. 1B and table S1). As expected (17, 18), these included the three subunits of eIF2 (α, β, γ), as well as PP1 (Fig. 1B and table S1). Notably, R15B^414-713^ also co-purified with all 5 subunits of eIF2B (α, β, γ, δ, ε) (Fig. 1B and table S1). This was confirmed by immunoblot analyses (fig. S1A). The paralog R15A also co-purified with eIF2, PP1 and eIF2B (fig. S1B). Furthermore, affinity purification of endogenously FLAG-tagged R15B also yielded eIF2, PP1 and eIF2B, establishing the biological relevance of the findings (fig. S1C).

The eluate from the R15B^414-713^ immunoprecipitation separated on size exclusion chromatography (SEC) in two distinct peaks: A large complex composed of R15B, eIF2 and eIF2B and a small complex containing R15B and eIF2 (Fig. 1C and D and table S2). PP1 was detected in both complexes (Fig. 1D and table S2). This showed that R15B bound both free eIF2 and eIF2 bound to eIF2B. This suggested that this eIF2-eIF2B complex might be a physiological substrate of the eIF2 phosphatases.

These findings revealed both a problem and its solution. The substrate of eIF2 phosphatases was previously thought to be free P-eIF2 (14–16, 19). However, it is not the abundance of P-eIF2 per se that dictates the rates of translation initiation, but the availability of rate-limiting eIF2B, which is sequestered in an inhibited state upon binding to P-eIF2 (Fig. 1E). Thus, unless eIF2B is rescued from P-eIF2, translation initiation remains inhibited. We then took a structural and biochemical approach to evaluate the possible function of R15B in this process.

## Structure of a native eIF2-eIF2B complex bound to R15B revealed GTP bound to eIF2Bγ

**Fig. 2:**
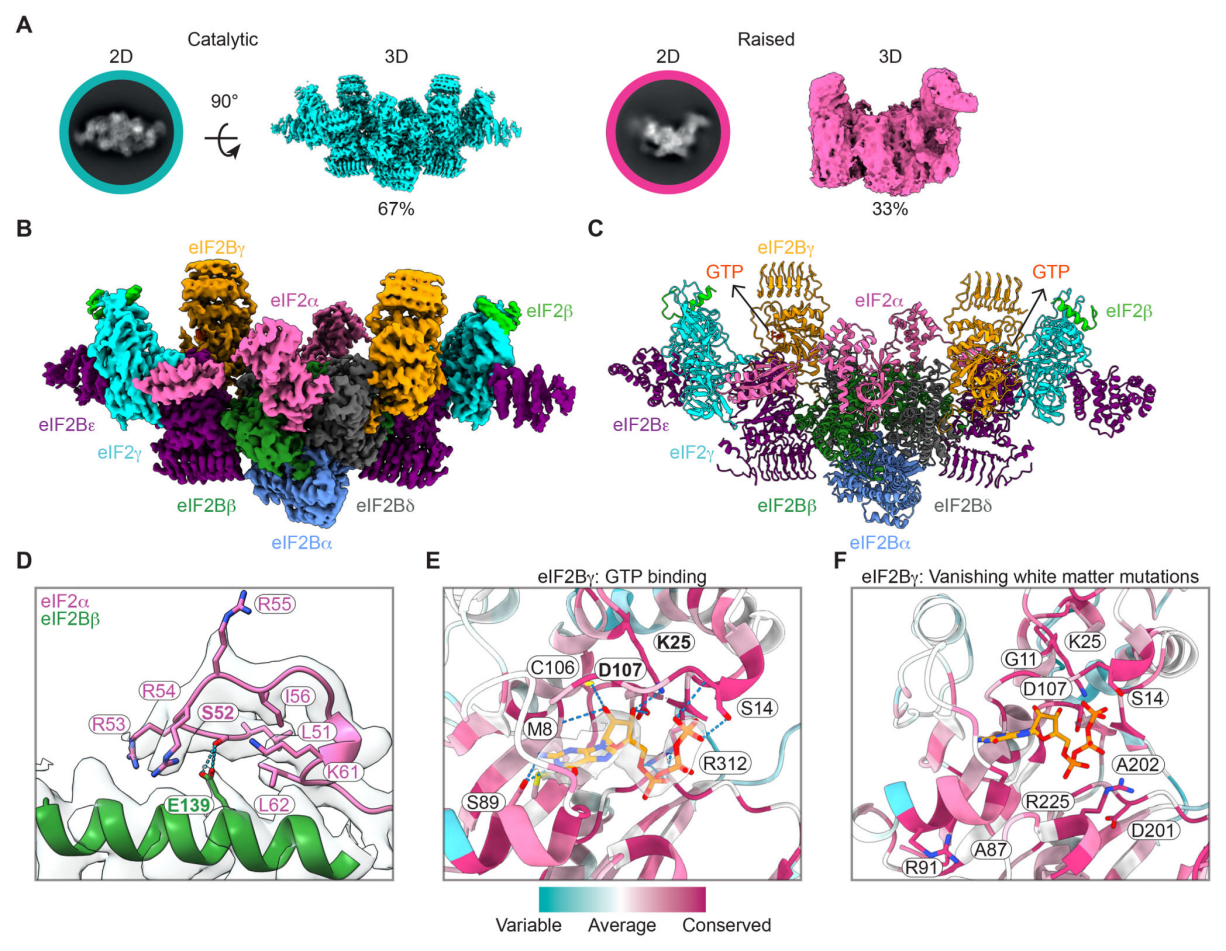
Cryo-EM map of native eIF2-eIF2B catalytic complex revealed GTP bound to eIF2Bγ. (**A**) Representative 2D classes and 3D cryo-EM reconstructions of eIF2-eIF2B complexes bound to R15B^414-713^ in catalytic (2.7 Å overall resolution) and raised conformations (3.8 Å overall resolution). (**B**) Cryo-EM map of eIF2-eIF2B in catalytic conformation (2.7 Å overall resolution). eIF2Bα (navy blue), eIF2Bβ (dark green), eIF2Bγ (yellow), eIF2Bδ (grey), eIF2Bε (purple), eIF2α (pink); eIF2β (lime); eIF2γ (cyan). R15B was invisible. (**C**) Structure of eIF2-eIF2B. R15B not modelled. (**D**) Close up view of the S52 loop of eIF2α forming two hydrogen bonds (blue) with the sidechain carboxyl group of eIF2Bβ E139. (**E** and **F**) Close up views of GTP bound to eIF2Bγ. Residues are coloured from cyan (variable) to burgundy (conserved) according to Consurf conservation score (52). (**E**) Density for GTP in the GTP-binding pocket. Residues forming hydrogen bonds (blue) with GTP are shown with sidechains. K25 and D107 (yeast K66 and D173, respectively) are in bold. (**F**) Vanishing white matter mutations mapping to the GTP-binding pocket of eIF2Bγ are indicated.

While the small R15B-eIF2 complex could not be structurally studied, possibly due to high flexibility, the large complex was resolved by single particle electron cryo-microscopy (cryo-EM) analysis (fig. S2 and table S3). 2D and 3D classification revealed recognizable features of eIF2-eIF2B complexes (Fig. 2A). Excluding low resolution classes, 67% of 3D particles displayed typical features of catalytic eIF2-eIF2B complexes (Fig. 2A and fig. S2). The remaining 33% of 3D particles exhibited a distinct, raised conformation that didn’t reach high resolution (Fig. 2A and fig. S2). Non-uniform refinement of the catalytic particles resulted in a structure of eIF2-eIF2B with an overall resolution of 2.7 Å (Fig. 2, B and C). eIF2B was a two-fold symmetric heterodecamer: two copies of eIF2B α, β and δ formed the core and eIF2Bγ and the catalytic subunit eIF2Bε flanked each side (Fig. 2, B and C). The two catalytic sites of this eIF2B decamer were bound to eIF2 (Fig. 2 B and C). The N-terminus of eIF2α bound an interface between eIF2Bβ and eIF2Bδ and eIF2γ was sandwiched between the core and the GEF domains of eIF2Bε (Fig. 2, B and C). This native complex resembled catalytic complexes prepared with recombinant eIF2 and eIF2B (fig. S3A), with eIF2B in the active state (fig. S3B) (12, 13, 21). S52 of eIF2α was resolved and unphosphorylated, forming hydrogen bonds with each sidechain oxygen of E139 of eIF2Bβ (Fig. 2D).

The nucleotide pocket of eIF2γ was empty (fig. S3C). eIF2γ coordinates nucleotide binding via a series of G-motifs: G4 and G5 coordinate the nucleotide base, G1/P-loop coordinates the phosphates (22) and the conformation of G2/switch loop 1 and G3/switch loop 2 defines the GTP or GDP bound states (23). The diameter of the P-loop in our eIF2γ structure was 5.3 Å, too narrow to accommodate a nucleotide (fig. S3, C and D). The absence of GDP and the conformation of eIF2γ in this eIF2-eIF2B catalytic complex suggested that GDP dissociation from eIF2γ precedes GTP loading.

The binding sites of previously reported chemical or natural activators of eIF2B, ISRIB and sugar phosphates (24, 25), were also unoccupied in this native complex (fig. S3, E and F). Notably, we found a GTP molecule bound to each eIF2Bγ in the native eIF2-eIF2B complex (Fig. 2C). This was not observed with previous structures of recombinant human eIF2-eIF2B nor yeast eIF2-eIF2B complexes (12, 13, 26, 27) although GTP can bind and activate purified eIF2B in vitro (28–30). eIF2Bγ bound GTP via several highly conserved residues in its N-terminal domain (Fig. 2E). Two eIF2B loss-of-function mutants were reported in residues K66 and D173 of yeast eIF2Bγ (29), corresponding to human residues K25 and D107 respectively. These directly bound GTP in our native eIF2-eIF2B complex, each forming a hydrogen bond with a hydroxyl group in the pentose sugar of GTP (Fig. 2E).

## The GTP-binding pocket of eIF2Bγ is a hotspot for mutations in vanishing white matter disease

In humans, mutations in eIF2B subunits cause vanishing white matter disease (9) by unclear mechanisms (31). We found that a third of the reported *EIF2B3* (encoding eIF2Bγ) pathogenic missense mutations mapped to the GTP-binding pocket (Fig. 2F). The pathogenic mutations G11V, S14P, S14F, K25R, D107N, R225Q, R225P (32) affected highly conserved residues that directly contacted the GTP (Fig. 2F). Four other disease-causing mutations, A87V, R91H, D201G, A202T (32) also mapped to highly conserved residues that formed the scaffold of the GTP-binding pocket (Fig. 2F). Thus, missense mutations associated with vanishing white matter disease mapped to the conserved GTP-binding pocket of eIF2Bγ.

This structure of endogenous eIF2-eIF2B revealed detailed native features. However, no densities for R15B were observed. Our previous study identified interaction sites between recombinant R15B and recombinant eIF2 (18). In the native structure, these sites on eIF2 were either bound to eIF2Bε, inside eIF2γ and thereby inaccessible, or devoid of densities (fig. S3G). Thus, in this eIF2-eIF2B complex, R15B was not bound to its predicted site but to an alternative site, unresolved probably because of high flexibility. Identifying R15B by cryo-EM was expected to be difficult because it is mostly unstructured (18).

## Structure of a native R15B-P-eIF2-eIF2B complex

**Fig. 3:**
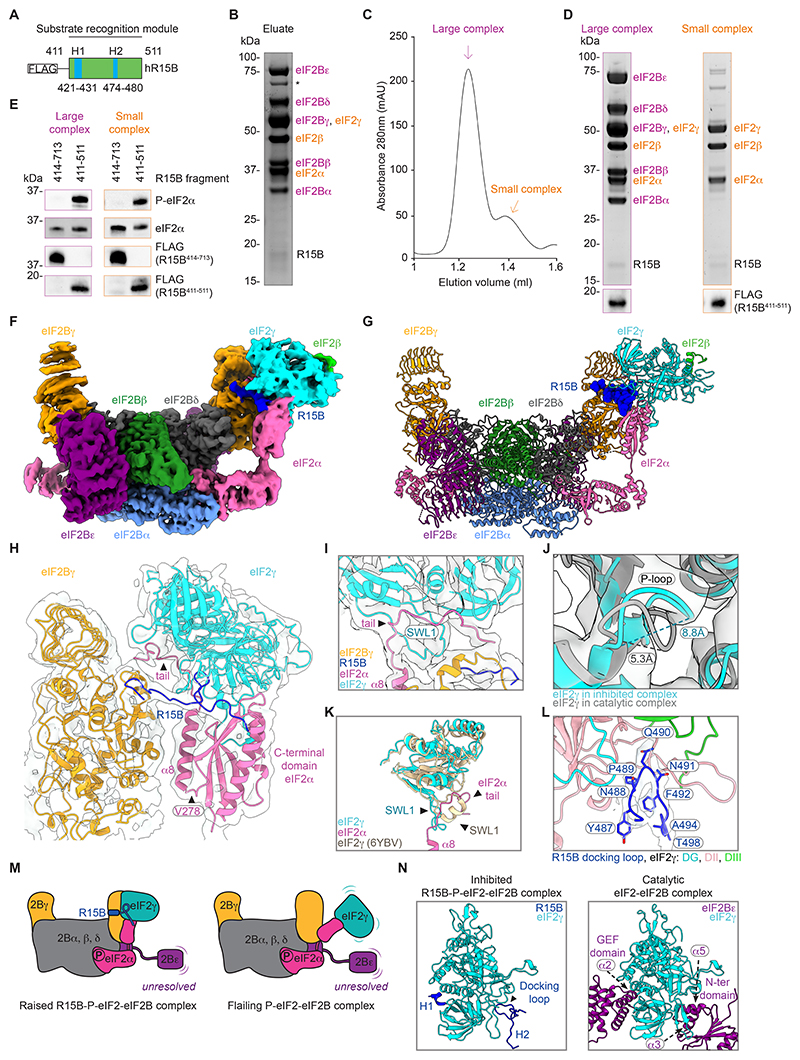
R15B tethers eIF2γ to eIF2Bγ in a P-eIF2-eIF2B complex. (**A**) Cartoon of R15B^411-511^ fragment used for purification. (**B**) Coomassie-stained gel of FLAG-R15B^411-511^ immunopurification. (**C**) Representative SEC of FLAG-R15B^411-511^ eluate (B). (**D**) Coomassie-stained gel of peak fractions from SEC (C). Immunoblots with FLAG antibody (bottom). (**E**) Immunoblots of indicated proteins in the large and small complex fractions from FLAG-R15B^414-713^ and FLAG-R15B^411-511^ immunopurifications. (**F**) Composite cryo-EM map of the R15B^411-511^-P-eIF2-eIF2B inhibited complex. Subunits coloured as in Fig. 2B. R15B (dark blue). (**G**) Composite structure of (F). R15B shown as spheres. (**H**) Close up view of R15B forming a tether between eIF2Bγ and eIF2γ and of helix α8 and the C-terminal tail of eIF2α, previously unresolved. (**I**) Close up view of the C-terminal tail of eIF2α and switch loop 1 (SWL1) of eIF2γ in an open conformation. (**J**) Alignment of eIF2γ in the inhibited complex (blue and cryo-EM density) with eIF2γ in the catalytic complex (grey), close-up view of the P-loop. (**K**) Alignment of eIF2γ in the inhibited R15B-P-eIF2-eIF2B complex (blue) with eIF2γ in pre-initiation complex (6YBV) (beige). (**L**) Close-up of R15B docking loop density (P486-T498) and eIF2γ. (**M**) Cartoon depicting the raised conformation of R15B^411-511^-P-eIF2-eIF2B compared to a recombinant P-eIF2-eIF2B complex (13) (**N**) Binding of R15B and eIF2Bε to eIF2γ in the inhibited (left) and catalytic (right) complexes.

The remaining 33% of R15B^414-713^ particles (Fig. 2A) resembled recombinant inhibited P-eIF2-eIF2B complexes (fig. S3H) with a notable distinguishing feature regarding the position of eIF2: eIF2γ was raised against the body of eIF2B in our R15B-bound complex, whereas it flails out in a recombinant P-eIF2-eIF2B complex (13) (fig. S3H). We thus refer to this native complex as the raised conformer. Although it could not be resolved to high resolution (fig. S2), we suspected that this conformation was caused by R15B binding to the P-eIF2-eIF2B complex and aimed to purify it by enriching for P-eIF2. Based on our previous work (17, 18), we designed a minimized R15B substrate-trap fragment, R15B^411-511^, which contained key eIF2-binding helices H1 (421-431) and H2 (474-480) (18) (Fig. 3A). FLAG-R15B^411-511^ immunopurified with the 3 subunits of eIF2 and the 5 subunits of eIF2B (Fig. 3B). The SEC profile of the R15B^411-511^ eluate revealed that it was predominantly composed of the large R15B-eIF2-eIF2B complex (Fig. 3, C and D, and tables S4 to S6). As expected, the levels of P-eIF2α were increased compared to that of complexes purified with dephosphorylation-competent R15B^414-713^ (Fig. 3E). Thus, P-eIF2 bound to eIF2B may indeed be a relevant substrate for its phosphatase.

Single particle cryo-EM analyses of R15B^411-511^-P-eIF2-eIF2B (fig. S4 and table S3) revealed a complex in the raised conformation that reached an overall resolution of 3.0 Å, 2.5 Å for the core and 6 Å for the periphery (fig. S4 and table S3). We used focused refinements on the most mobile parts of the complex, eIF2Bγ and eIF2γ (fig. S4), increasing their local resolution to 3.8 Å (Fig. 3F, fig. S4 and table S3). The structure of this R15B-P-eIF2-eIF2B complex obtained with R15B^411-511^ fitted well with the raised particles purified with R15B^414-713^ (fig. S5A). In the R15B^411-511^-P-eIF2-eIF2B structure, eIF2B was in the typical inhibited state (fig. S5B) (12, 13, 21), bound to two eIF2 trimers, one of which could be resolved (Fig. 3, F and G). The N-terminus of eIF2α bound an interface between eIF2B α and eIF2Bδ (Fig. 3, F and G), defined as the inhibitory site (12, 13), as expected for P-eIF2. The C-terminal domain of eIF2α was in a raised position against eIF2Bγ (Fig. 3 F and G). In this conformation, the previously unresolved C-terminal helix (α8) and tail of eIF2α (12, 13) were visible (Fig. 3H). Helix α8 extended upwards towards eIF2γ, ending in a tail sandwiched between eIF2γ and eIF2Bγ (Fig. 3 H and I). The resolution of eIF2γ was not sufficient to resolve nucleotide binding. However, we could examine its binding pocket. The G domain of eIF2γ in the R15B^411-511^-P-eIF2-eIF2B complex aligned well (RMSD: 1.34 Å) with the crystal structure of archaeal IF2γ bound to GDP (1KK3) (23) (fig. S5C). Moreover, the diameter of the P-loop (G1) of eIF2γ in the inhibited complex was 8.8 Å compared to 5.3 Å in the nucleotide-free eIF2γ of the catalytic complex (Fig. 3J). Additionally, switch loop 1 of eIF2γ was in an open conformation (Fig. 3I). Together, these structural features established that eIF2γ adopted a GDP-bound conformation in the inhibited R15B^411-511^-P-eIF2-eIF2B complex. When bound to GTP, switch loop 1 in eIF2γ changes conformation, folding into an α-helix as seen in the pre-initiation complex (33). The C-terminal tail of eIF2α blocked this conformational change in the R15B^411-511^-P-eIF2-eIF2B complex (Fig. 3K). This implies that the C-terminal tail of eIF2α, holds eIF2γ in a GDP-bound state.

## R15B forms a tether between eIF2Bγ and eIF2γ in a native R15B-P-eIF2-eIF2B complex

A defining feature of the R15B^411-511^-P-eIF2-eIF2B complex was the position of eIF2γ in a raised conformation against eIF2Bγ (Fig. 3, F and G). This was not due to direct contacts between eIF2Bγ and eIF2γα but to a density between these subunits, identified as R15B (Fig. 3, H and L, fig. S5 D to F and table S3). R15B H2 bound the N-terminal domain of eIF2Bγ (Fig. 3H and fig. S5D). Downstream of H2, R15B extended and docked with residues P486-A494 between all three domains of eIF2γ, forming a docking loop (Fig. 3, H and L). At its apex, Q490, pointed towards eIF2γ domains G and II (Fig. 3L). Downstream of A494, R15B extended towards the C-terminus of eIF2α (Fig. 3H). This shows that R15B formed a tether that held eIF2γ, and with it the interacting C-terminus of eIF2α, in a raised conformation against eIF2Bγ (Fig. 3, F to H). Consequently, the flexible loop linking the C- and N-terminal domains of eIF2α was rigid (Fig. 3, F and G). We subsequently used the raised conformation as the structural signature of the native R15B-P-eIF2-eIF2B complex (Fig. 3M).

Our previous study with recombinant R15B and eIF2 revealed that H1 of R15B is critical for recruiting free eIF2 (18). An extra density binding the G-domain and domain III of eIF2γ was observed in the native R15B^411-511^-P-eIF2-eIF2B complex (fig. S5E). Docking an AlphaFold2 model of R15B (predicted in the context of eIF2), fitted R15B H1 to this unassigned density (fig. S5E). This ideally positioned the negatively charged region of R15B between H1 and H2 over a positively charged surface of eIF2γ (fig. S5F). This model explains the anchoring function of H1 that, in combination with H2 and the docking loop, forms a clamp to grasp the eIF2γ subunit of eIF2, approximately 107 Å away from the target P-S52 on eIF2α. Thus, R15B uses the same binding regions to capture both free and eIF2B-bound eIF2.

Like in the catalytic complex (fig. S3, E and F), the binding sites of the previously reported chemical or natural activators of eIF2B, ISRIB and sugar phosphates (24, 25), were also empty in this native complex (fig. S5, G and H). We then mapped the binding sites of R15B resolved in the inhibited complex to the catalytic complex. In the catalytic complex, the position of R15B H1 and its docking loop on eIF2γ were occupied by helix α2 of eIF2Bε’s GEF domain and helices α3 and α5 of eIF2Bε’s N-terminal domain respectively (Fig. 3N). The R15B H2 binding site on the N-terminal domain of eIF2Bγ was empty in the catalytic complex (fig. S5I). This has two implications. First, the binding of eIF2γ to eIF2Bε (the catalytic subunit of eIF2B) and to R15B is mutually exclusive. Second, the R15B-binding sites on the inhibited complex are either occupied or empty in the catalytic complex. Thus, R15B binds two different sites in the catalytic and inhibited eIF2-eIF2B complexes, the latter of which could be resolved.

## Mutants of R15B with reduced binding to the eIF2B-eIF2 complex

**Fig. 4:**
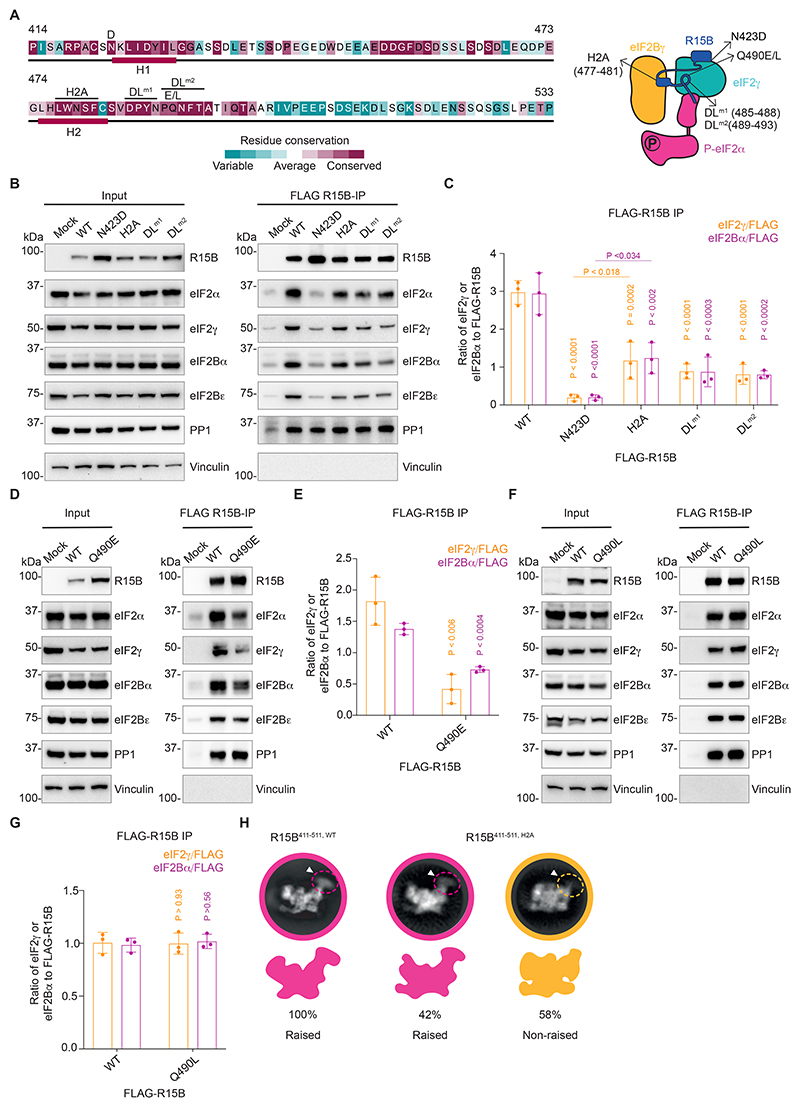
Mutations of R15B destabilize the R15B-eIF2-eIF2B complex and the raised conformation. (**A**) Sequence conservation of R15B^414-533^ according to Consurf (52) with mutations shown above the sequence (left). H2, docking loop (DL) mutant (m) 1 and 2 were replaced by alanines. Cartoon depicting the positions of the mutations (right). (**B**) Inputs and eluates of immunoprecipitated complexes revealed by immunoblotting with indicated antibodies. Representative results of n=3. (**C**) Quantification from (B). Data are means ± SD. (n= 3). One-way ANOVA with Tukey’s multiple comparisons test. (**D, E**) Same as (B, C) with R15B Q490E mutant. Unpaired t-test. (**F, G**) Same as (D, E) with R15B Q490L mutant. (**H**) Representative 2D classes of R15B^411-511^-P-eIF2-eIF2B complexes purified with wild-type (left) or H2A mutant (right) R15B^411-511^. White arrowhead and dashed circles indicate the density corresponding to eIF2γ. Full sets of immunoblots are presented in fig. S6.

We next used mutagenesis to test the functional significance of the R15B features observed in the R15B^411-511^-P-eIF2-eIF2B complex. First, we examined a previously reported variant of R15B, R15B^N423D^, in a residue adjacent to H1 (Fig. 4A). This variant reduces binding to eIF2 and causes a rare syndrome with microcephaly, developmental delay and intellectual disability (18). We found that capture of eIF2B by R15B^N423D^ was severely compromised (Fig. 4 B and C and fig. S6A). This confirms the importance of this region in binding both free and eIF2B-bound eIF2 (fig. S5E).

We next turned to the H2A mutant (18) with alanines replacing H2 residues 477-481 (Fig. 4A). R15B^H2A^ captured less eIF2 and eIF2B than wild-type R15B (Fig. 4 B and C and fig. S6A). We then replaced the first or second half of the docking loop with alanines (Fig. 4A). Both mutants captured significantly less eIF2 and eIF2B compared to wild-type R15B (Fig. 4 B and C and fig. S6A). The H2A and docking loop mutants retained PP1 binding (Fig. 4B) because they contained the PP1-binding region.

Next, we generated R15B^Q490E^ to assess the importance of Q490 at the apex of the docking loop (Fig. 3L and 4A). R15B^Q490E^ was severely compromised in capturing both eIF2 and eIF2B (Fig. 4 D and E and fig. S6B). In contrast, R15B^Q490L^ retained wild-type properties (Fig. 4 F and G and fig. S6C). As anticipated, the R15B^Q490E/L^ mutations did not affect PP1 binding (Fig. 4 D and F). Thus, the apex of the docking loop is key for capturing the eIF2-eIF2B complex and introduction of a bulky negatively charged sidechain at R15B residue 490 is incompatible with the largely uncharged pocket of eIF2γ into which Q490 extends. These mutagenesis experiments validate the importance of the structural features of R15B for binding eIF2-eIF2B and further support the identification of this complex as a physiological substrate for R15B.

The binding of R15B H2 to eIF2Bγ appeared critical for defining the raised conformation of eIF2γ in the R15B^411-511^-P-eIF2-eIF2B complex. Thus, we examined the consequence of the H2A mutation on the raised conformation by cryo-EM 2D-class analysis (Fig. 4H, fig. S7 and tables S7 and S8). All the 2D classes obtained from R15B-P-eIF2-eIF2B particles purified with wild-type R15B^411-511^ were in a raised conformation (Fig. 4H). In contrast, only 42% of H2A mutant R15B^411-511^ 2D classes retained the raised conformation (Fig. 4H). This demonstrates that R15B H2 is a critical part of the tether holding P-eIF2 in the raised conformation against eIF2B.

## Function of R15B when bound to the P-eIF2-eIF2B complex

**Fig. 5:**
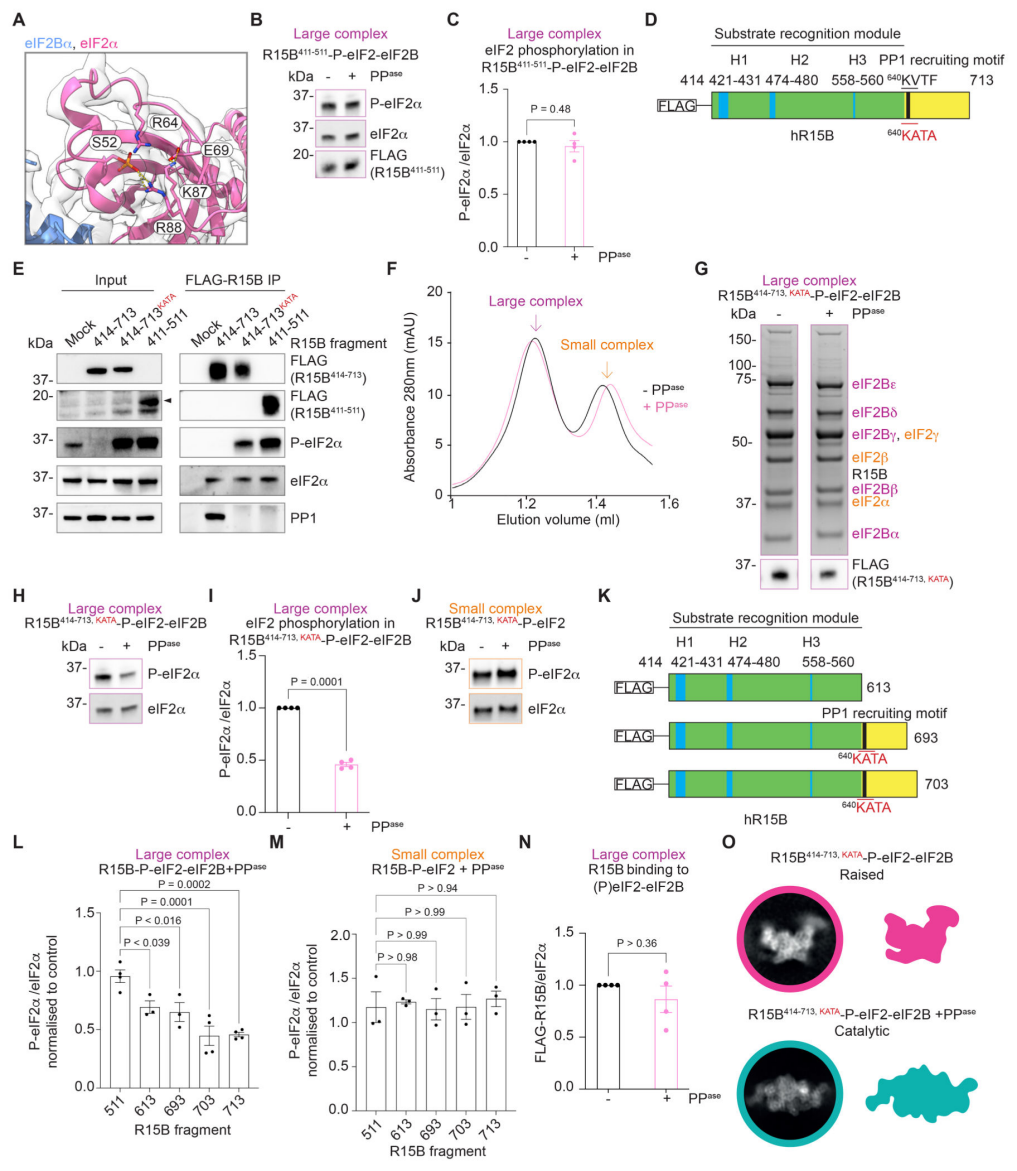
R15B rescues eIF2B from P-eIF2. (**A**) Close-up of P-S52 of eIF2α trapped by intramolecular salt-bridges in the R15B^411-511^-P-eIF2-eIF2B inhibited complex. (**B**) Immunoblots of indicated proteins in R15B^411-511^-P-eIF2-eIF2B complexes with or without alkaline phosphatase (AP) treatment. (**C**) Ratio of P-eIF2α to eIF2α in samples from (B). Data are means ± SEM (n=4), paired t-test. (**D**) Cartoon of N-terminal FLAG-tagged R15B^414-713, KATA^ fragment. (**E**) Representative immunoblots of indicated proteins from inputs and eluates immunopurified with indicated FLAG-R15B fragments (n=2). (**F**) Representative SEC profiles of FLAG-R15B^414-713, KATA^ complexes without (black) or with (pink) AP treatment, n>3. (**G**) Representative Coomassie-stained gels of R15B^414-713, KATA^-P-eIF2-eIF2B complexes from (F) (top) and FLAG immunoblots (bottom). (**H, J**). Representative immunoblots of indicated proteins in large (H) or small (J) complexes with or without phosphatase treatment.(**I**) Ratio of P-eIF2α to eIF2α in samples from (H). Data are means ± SEM (n=4), paired t-test. (**K**) Cartoons of R15B truncations. (**L, M**) Quantification of eIF2α dephosphorylation in R15B-P-eIF2-eIF2B (L) or R15B-P-eIF2 (M) complexes immunopurified with indicated R15B fragments. See also (fig. S8 B to E). Data are means ± SEM (n≥ 3), one-way ANOVA with Dunnett’s multiple comparisons test. (**N**) Ratio of R15B to eIF2α in large complexes purified with R15B^414-713, KATA^ and with or without AP treatment. Data are means ± SEM (n=4), paired t-test. (**O**) Representative 2D classes and cartoons of R15B^414-713, KATA^-P-eIF2-eIF2B with and without phosphatase treatment.

Until now the mechanism by which eIF2B is rescued from P-eIF2 inhibition has been unclear. Our findings suggested that R15B might enable dephosphorylation of P-eIF2 on eIF2B. First, we examined the phosphorylation status of eIF2α in the R15B^411-511^-P-eIF2-eIF2B complex. The resolution of the cryo-EM map revealed that S52 of eIF2α was phosphorylated (Fig. 5A). The phosphate group formed two salt bridges with the two sidechain amine groups of R88 in eIF2α (Fig. 5A). The loop containing S52 was further stabilized by two additional salt bridges between the sidechain amine group of K87 and the sidechain carboxyl group of E69 (Fig. 5A). Thus, P-S52 was trapped by intramolecular electrostatic interactions in this R15B^411-511^-P-eIF2-eIF2B complex, as in recombinant P-eIF2-eIF2B complexes lacking R15B (12, 13). This suggested that the complex was dephosphorylation-incompetent. Indeed, we probed the complex with a heterologous phosphatase and found that P-eIF2α was resistant to dephosphorylation (Fig. 5, B and C). In contrast, recombinant P-eIF2α was readily dephosphorylated (fig. S8A). This demonstrates that P-eIF2 is inaccessible for dephosphorylation in the R15B^411-511^-P-eIF2-eIF2B complex. Thus, if R15B were to rescue eIF2B from P-eIF2 inhibition, the R15B^411-511^ fragment was unable to do so.

We next returned to the longer C-terminal fragment R15B^414-713^ and mutated the canonical PP1-recruitment site (KVTF to KATA) (34, 35) to reduce PP1 binding (Fig. 5D) and trap P-eIF2. FLAG-R15B^414-713^ recruited PP1 to form a functional holoenzyme that dephosphorylated P-eIF2, thereby reducing basal levels of eIF2α phosphorylation in cell lysates (Fig. 5E). In contrast, FLAG-R15B^414-713, KATA^ failed to recruit PP1 and increased basal phosphorylation levels of eIF2α in cell lysates (Fig. 5E), similar to the substrate-trapping FLAG-R15B^411-511^ fragment (Fig. 5E). Phosphorylation of eIF2α was increased relative to wild-type FLAG-R15B^414-713^ in complexes immunopurified with FLAG-R15B^414-713, KATA^ and FLAG-R15B^411-511^ (Fig. 5E). Thus, mutating the PP1-binding site of R15B^414-713^ trapped P-eIF2.

We then examined if the R15B^414-713, KATA^ fragment could enable dephosphorylation of the otherwise dephosphorylation-incompetent P-eIF2-eIF2B complex. We subjected immunopurified R15B^414-713, KATA^ complexes to phosphatase treatment followed by SEC. Notably, the SEC profile of the phosphatase-treated eluate was almost identical to that of the untreated R15B^414-713, KATA^ eluate (Fig. 5, F and G). However, dephosphorylation of the large R15B^414-713, KATA^-P-eIF2-eIF2B complex was effective (Fig. 5, H and I). In contrast, the small complex was not dephosphorylated (Fig. 5J). Thus, R15B^414-713, KATA^ enables P-eIF2 dephosphorylation on eIF2B.

We next generated a series of C-terminal deletions of R15B and assessed their ability to enable dephosphorylation of P-eIF2 on eIF2B (Fig. 5, K and L and fig. S8 C to E), incorporating the KATA mutation where necessary (Fig. 5K). Dephosphorylation of the large R15B-P-eIF2-eIF2B complex progressively decreased upon deletion of the C-terminus of R15B (Fig. 5L and fig. S8 C to E). As observed with R15B^414-713, KATA^, the small complex was not dephosphorylated in the different conditions (Fig. 5M and fig. S8 B to E). Thus, the C-terminal region of R15B is required for dephosphorylation of P-eIF2 on eIF2B.

We next examined the fate of dephosphorylated eIF2. We expected that dephosphorylation of P-eIF2 bound to eIF2B would release eIF2. However, the abundance of the small complex did not increase after dephosphorylation on the large complex in the SEC profile (Fig. 5F and fig. S8 B to E), indicating that dephosphorylated eIF2 did not permanently dissociate from eIF2B. Furthermore, R15B also remained largely bound to the eIF2-eIF2B complex after dephosphorylation (Fig. 5, G and N). We wondered if dephosphorylation changed the conformation of the complex and turned to single particle cryo-EM 2D classification. All the 2D classes of untreated R15B^414-713, KATA^-P-eIF2-eIF2B complexes displayed the raised conformation (Fig. 5O), similar to R15B^411-511^-P-eIF2-eIF2B complexes (Fig. 4H). This confirmed that untreated R15B^414-713, KATA^-P-eIF2-eIF2B complexes were in the inhibited state (Fig. 5O). Remarkably, the 2D classes of the phosphatase-treated R15B^414-713, KATA^-P-eIF2-eIF2B complexes were all in catalytic conformation (Fig. 5O). Thus, phosphatase treatments switched the R15B^414-713, KATA^-P-eIF2-eIF2B complexes from the inhibited to the catalytic conformation. This indicates that dephosphorylation of eIF2 released it from the inhibitory site and allowed its transfer to a catalytic site. Thus, R15B renders P-S52 of eIF2α accessible to dephosphorylation on the eIF2B complex, thereby rescuing eIF2B from P-eIF2 inhibition.

## Discussion

The dynamic and reversible phosphorylation of eIF2α, also known as ISR, has been studied for several decades (1, 2) but its termination has remained elusive. All the prior work focused on free P-eIF2 as the substrate of its phosphatases. Here we show that R15B binds to a P-eIF2-eIF2B complex and enables dephosphorylation of P-eIF2 on eIF2B, thereby rescuing both eIF2 and eIF2B for translation (fig. S9).

Structural analyses of R15B bound to P-eIF2-eIF2B complexes revealed that R15B^411-511^ not only contacts P-eIF2, but also engages eIF2B directly, forming a tether between eIF2γ and eIF2Bγ to position P-eIF2 in a raised conformation on eIF2B. In addition to recruiting the substrate, we show here that R15B enables its dephosphorylation on eIF2B. This is carried out by R15B’s C-terminus, probably by inducing a conformational change that leads to the exposure of otherwise inaccessible P-S52 in eIF2α. Thus, R15B fulfills two important functions in addition to recruiting PP1: It recruits the substrate and primes it for dephosphorylation.

Once dephosphorylated, P-eIF2 is released from the inhibitory site of eIF2B and transfers to one of its catalytic sites. This allows the conversion of the inhibitor into a substrate on eIF2B. We favor the idea that dephosphorylated eIF2 translocates from the inhibitory to a catalytic site on the same eIF2B, as opposed to a neighboring free eIF2B, because of the proximity of two catalytic sites with subnanomolar affinity for eIF2 within the same eIF2B decamer (36). Intramolecular transfer would also ensure concomitant reactivation of eIF2B, regardless of the remaining pool of P-eIF2. Notably, P-eIF2 bound to R15B was dephosphorylation-incompetent in our assay. Knowing that eIF2 is highly dynamic and can switch from open to close conformation (37), R15B may capture free P-eIF2 and maintain it in a closed conformation, preventing its dephosphorylation until bound to eIF2B, thereby coupling dephosphorylation to reactivation. Indeed, translation recovery can be observed before full dephosphorylation of eIF2 (38).

We found that R15B bound to eIF2-eIF2B complexes regardless of the phosphorylation state of eIF2. However, R15B occupies two distinct sites: one in the inhibited complex and another in the catalytic one. The unstructured nature of R15B might be a key feature to allow this plasticity between the two sites.

Structural analyses of the inhibited R15B-P-eIF2-eIF2B complex revealed an additional function of R15B: It contributes to hold the complex in an inhibited state in three ways. The tether that R15B forms between eIF2γ and eIF2Bγ holds eIF2γ in a raised conformation, where it cannot flail out to engage the catalytic eIF2Bε. Moreover, the binding of R15B and eIF2Bε to eIF2γ is mutually exclusive. Thus, R15B prevents GEF activity in this complex. Furthermore, R15B binding to the P-eIF2-eIF2B complex stabilized the C-terminus of eIF2α. This in turn blocks the conformational change required for eIF2γ to bind GTP, holding it in a GDP-bound conformation. This is reminiscent of a GDP-dissociation inhibitory function. By contributing to the inhibited state of the P-eIF2-eIF2B complex and promoting dephosphorylation of eIF2 on eIF2B, R15B couples dephosphorylation to reactivation of both eIF2 and eIF2B. There might be an additional level of regulation, perhaps in the recruitment of PP1.

Moreover, we found that human native eIF2Bγ binds GTP in a conserved pocket, a hotspot for vanishing white matter disease mutations, highlighting the importance of working with native complexes to uncover biochemical features of disease relevance.

This work reveals the feasibility of studying the structure and dynamic function of largely unstructured non-catalytic subunits of phosphatases bound to their large substrates. There are more than 200 non-catalytic subunits of PP1, often with large unstructured regions (34, 35), that could be studied in a similar way.

The power and benefit of homeostatic translation regulation by phosphorylation of eIF2α lies in its dynamic reversibility to provide cells with rapid adaptation to changes in their environment. Here we showed that R15B rescues eIF2B from P-eIF2 inhibition. This enables eIF2 and eIF2B to re-enter the translation initiation cycle, thereby preventing the lethal consequences of persistent translation inhibition.

## Materials and methods

### Mammalian cells

Expi293 (RRID: CVCL_A784) suspension cells were grown in Expi293™ expression medium (Thermo Fisher, A14351) in 850 cm^2^ roller bottles (Corning, 430849) in a humidified incubator at 37°C, 8% CO_2_, 125 RPM and kept at densities between 0.5 to 2x10^6^ cells/ml in a maximum volume of 600 ml per 850 cm^2^ bottle during maintenance.

Human embryonic kidney 293T cells (HEK293T) (RRID: CVCL_0063) and HeLa (RRID: CVCL_0030) cells were grown at 37°C with 5% CO2 in a humidified incubator. Both were grown in Dulbecco’s Modified Eagle’s Media (DMEM, Sigma, D5796) supplemented with 10% foetal bovine serum (FBS, Gibco 10270), 2 mM L-glutamine (Gibco, 25030), 100 U/mL penicillin and 100 μg/mL streptomycin (Gibco, 15140122).

### Cloning

R15 constructs were cloned into the mammalian expression vector PXJ41 (39), which contains a single N-terminal FLAG tag, using the Gibson Assembly method (40). R15B poly-alanine mutants (477-481 (H2A), 485-488 (DL^m1^), 489-493 (DL^m2^)) were ordered as GeneArt String DNA fragments (Life Technologies Ltd). The R15B Q490E, Q490L and R15B KATA mutations were generated using QuickChange II Site-Directed mutagenesis kit (Agilent, 200523) following manufacturer’s instructions. R15B N423D mutant was generated in (18) using QuickChange II Site-Directed mutagenesis kit (Agilent, 200523).

### Generation of endogenously 3x-FLAG tagged R15B using the CRISPR-Cas9 system

The sgRNA plasmid pairs and donor plasmid were designed as follows. The donor plasmid pMA contains a GAGAGAGAGA linker prior to the 3x-FLAG which is inserted at the C-terminus of endogenous PPP1R15B. EGFP is encoded after 3x-FLAG preceded by an IRES2 element to enable fluorescence-activated cell sorting (FACS). The antisense guide was cloned into the Cas9 D10A vector pX335, the D10A Cas9 nickase minimizes off-target events. The sense guide was cloned into pBABED puro U6, enabling puromycin positive selection.

HeLa cells were seeded at 1.0x10^6^ cells per 10 cm^2^ dish and incubated for 24 hours. 1 μg sgRNA plasmid pairs and 3 μg of donor plasmid were mixed in 800 μl Opti-MEM and 20 μl PEI transfection reagent (Polysciences, 24765) and incubated for 20 min at RT, before dropwise addition to cells. Cells were incubated for 24 hours and then media was replaced with media containing 2 μg/ml puromycin. A second round of puromycin selection was conducted 24 hours later. Next, the puromycin containing media was replaced with fresh media for 8 hours before repeating the transfection detailed above. Following 60 hours of transfection, cells were prepared for FACS. 96-well plates were prepared containing 100 μl of fresh media. Cells were detached using trypsin, pelleted at 300 RCF and resuspended in filtered (sterile 50 μM filter, CellTrics, 04-004-2327) FACS media (sterile PBS, 1 % FBS, 2 mM EDTA) at a concentration of 1.0x10^6^ cells per ml. Cells were filtered by a sterile 50 μM filter into sterile polypropylene tubes.

Cells were sorted using a Bigfoot Spectral Cell Sorter (Thermofisher Scientific), calibrated for single cell sorting, using a 100 μm nozzle under sterile conditions. Cells were gated for intact single cells, before the top 1% of GFP-positive cells were selected using a 488 nm laser with a 507/19 nm filter. Selected cells were dispensed into the pre-prepared 96-well plates. Following FACS, single cells were grown for 2 weeks, expanded and analyzed for in-frame integration of the linker and 3x-FLAG tag by PCR, immunoblots and Sanger sequencing (Source Bioscience).

For immunoprecipitation experiments, 8x10cm^2^ dishes seeded at 1x10^6^ cells were used per condition.

### Transient transfection of HEK293T cells

HEK 293T cells were seeded at 0.8x10^6^ cells per 10 cm^2^ dish and incubated for 24 hours. Next, 4 μg of salmon sperm DNA (Invitrogen, 15632011V) (mock) or indicated R15 constructs were mixed with 800 μl of Opti-MEM media (Gibco, 11058) and 12 μl PEI transfection reagent (Polysciences, 24765) and incubated for 20 min at RT. The transfection mixture was subsequently added to cells in a dropwise manner, gently mixed and cells were incubated for a further 24 hours.

### Cell lysates

Cells were washed with 5 ml ice-cold PBS per dish and pelleted at 300 RCF for 5 min at 4 °C. Cell pellets were then lysed in 800 μl of lysis buffer (50 mM Tris-HCl pH 7.5, 150 mM NaCl, 0.5% triton, 10% glycerol, EDTA-free cOmplete protease tablet (Roche, 04693159001), phosstop (Roche, 04906837001)) while rotating at 4°C for 10 min. Lysates underwent microcentrifugation at 4 °C for 12 min at 16000 RCF and supernatants were transferred to fresh tubes. A 90 μl aliquot was taken for input samples, mixed with 30 μl 4X BOLT LDS (Novex #B0007) containing 100 mM DTT and boiled for 10 min at 95 °C.

### FLAG immunoprecipitation from HEK293T and HeLa cells

10 μl of anti-FLAG M2 magnetic beads (Sigma-Aldrich, M8823-1ML), pre-equilibrated in lysis buffer (as above), was used per 10cm^2^ dish of cells. Beads were added to 700 μl of lysates and incubated overnight while rotating at 4 °C. Samples were washed five times with lysis buffer (see above) prior to elution in 50 μl of 1X BOLT LDS (Novex #B0007) containing 100 mM DTT, boiled at 95 °C for 10 min. 10 μl of both lysates (input) and immunoprecipitated samples (FLAG-IP) were analyzed by immunoblots.

### Transient transfection of Expi293 cells

2x600 ml of cells at 2x10^6^ cells/ml were transfected transiently for protein complex purification. For 600 ml of 2x10^6^ cells/ml cells, 660 μg of FLAG-tagged R15B construct DNA was mixed with 1.78 mg of PEI_MAX_ and incubated in 40 ml Expi293™ expression medium for 15 min at room temperature before adding the mix to cells. Cells were transfected for 20-22 hours.

### FLAG-immunoprecipitation from transfected Expi293 cells

Cells were pelleted at 3000 RCF, 4 °C, 15 min. Cell pellets were washed with cold PBS, re-pelleted at 1200 RCF, 4 °C, 5 min and lysed in lysis buffer (50mM Tris-HCl pH 7.5, 150mM NaCl, 0.5% triton, 10% glycerol, EDTA-free cOmplete protease tablet (Merck, 11873580001), phosstop (Roche, 04906837001)) in a volume 4x the pellet volume for 10-15 min on ice, vortexing the lysate every 3 min. Lysates were then ultracentrifuged at 21,100 RCF, 4 °C, 10 min. After ultracentrifugation, the pellet was discarded and the supernatant incubated with either 500 μl Anti-FLAG M2 magnetic beads (Merck, M8823) or 280 μl Pierce™ Anti-DYKDDDDK Magnetic agarose (Thermo Fisher, A36798) per 10ml of cell pellet for 1 hour at room temperature. Before binding, beads were equilibrated in lysis buffer. After incubation, FLAG beads were washed 3x 5-10 min in cold lysis buffer and 3x 5-10 min in cold 1x TBS, at 4 °C. After washes, protein was eluted from beads in 150 μg/ml of 1x FLAG peptide (Merck, F3290) in 400-1000 μl of 1x TBS depending on bead amount and type used, at 4 °C or 30 °C for 45 min, 850 RPM on a temperature-controlled block. The eluate was analyzed by 4%–12% Bis-Tris Plus gel and stained with InstantBlue Coomassie protein stain (Abcam, ab119211). Eluate was then concentrated to 50 μl and analyzed by size-exclusion chromatography (SEC) by injection on a Superose 6 3.6/300 column (Cytiva) in an ÄKTA Pure system, equilibrated with SEC buffer (20 mM HEPES pH 7.5, 100 mM KCl, 1 mM TCEP). Fractions were then analyzed by 4%–12% Bis-Tris Plus gel and stained with InstantBlue Coomassie protein stain (Abcam, ab119211) or transferred for immunoblotting (see below). R15B-eIF2-eIF2B peak fractions in SEC buffer were used for cryo-EM without further manipulations.

### Phosphatase treatment of immunoprecipitated R15B complexes during FLAG elution

The anti-FLAG bead-bound complexes were aliquoted in two: One half was eluted with 150 μg/ml 1x FLAG peptide, 1x dephosphorylation buffer (from 10x dephosphorylation buffer, Merck, 10713023001). The other half was eluted with 150 μg/ml 1x FLAG peptide, 1x dephosphorylation buffer and 5 or 50 units of alkaline phosphatase (Merck, 10713023001) at 30 °C for 45 min. The eluate was then concentrated to 50 μl and injected to SEC for analysis as described above.

### Cryo-EM sample preparation and data collection

#### R15B^414-713^-eIF2-eIF2B complexes

Quantifoil 1.2/1.3 grids were glow discharged for 30 s at setting 6 using an Edwards S150B sputter coater discharger. 3 μl of sample in SEC buffer was applied to each grid. The grids were blotted using Whatman blotting paper (Grade 1) at a blot force of -3 for 3 s at 4 °C, 100% humidity and vitrified in liquid ethane using an FEI Vitrobot MK IV. Data collection was performed on a Thermo Fisher Titan Krios 3 transmission electron microscope operated at 300 kV and equipped with a Gatan K3 direct electron detector camera and a GIF Quantum energy filter. Automatic collection was performed using EPU software (Thermo Fisher), in counting mode at a physical pixel size of 0.824 Å per pixel, with a total electron dose of approximately 50 electrons per Å^2^ over a total exposure time of 3.0 s. Exposures were fractionated into 50 movie frames. Slit width of 20 eV on the energy filter and a defocus range of -1 to -3 μm were used. A total of 9971 movies were collected.

#### R15B^411-511^-P-eIF2-eIF2B complexes

UltrAuFoil 1.2/1.3 grids coated with 2 nm carbon film were glow discharged for 10 s using 15 mA at an Easiglow glow discharge system. 3 μl of sample was applied to each grid. The grids were blotted using Whatman blotting paper (Grade 1) at a blot force of -3 for 3 s at 4 °C, 100% humidity and vitrified in liquid ethane using a FEI Vitrobot MK IV. Data collection was performed on a Thermo Fisher Titan Krios 2 transmission electron microscope operated at 300 kV and equipped with a Falcon4i direct electron detector camera. Automatic collection was performed using EPU software (Thermo Fisher), in electron event recording mode at a physical pixel size of 1.1 Å per pixel, with a total electron dose of approximately 50 electrons per Å^2^. A defocus range of -0.8 to 2.4 μm was used. A total of 9438 movies were collected.

### Cryo-EM image processing

#### R15B^414-713^-eIF2-eIF2B complexes dataset

Movies were imported into Relion 5.0 (41) and motion corrected with Relion’s implementation of MotionCor using 7x6 patches and dose-weighting. The motion corrected micrographs were then CTF corrected with CTFFIND4 (42). Particle picking was performed on the CTF corrected micrographs using Topaz (43) with a pre-trained agent. 1248346 particles were extracted with 400 pixels box size Fourier cropped to 100 pixels (bin4). Extracted particles went through two rounds of 2D classification with 200 classes for 30 iterations, each round using the old EM algorithm and fast subsets. 2D classes which showed secondary structure features were selected (642259 particles) for 3D classification using 6x classes and a 7.5 degree search angle. Two primary classes emerged showing distinct protein features. These were identified as catalytic (256k particles) and raised (129k particles) complexes. The raised complexes were re-extracted at unbinned pixel size and imported in cryoSPARC. Non-uniform refinement of the raised subclass led to an overall 3.8 Å resolution volume which was not processed further. Catalytic particles were 3D refined then put in a 3D classification without image alignment with 3x classes. The highest resolution class contained 171k particles, which were re-extracted at unbinned pixel size and imported into cryoSPARC. Non-uniform refinement using C2 symmetry of the imported catalytic particles yielded an overall 2.7 Å resolution reconstruction. To check whether both eIF2Bγ subunits in this native complex were bound to GTP, symmetry restraints were relaxed and the map was refined again using C1 symmetry. This revealed that each eIF2Bγ subunit was bound to a molecule of GTP.

#### R15B^411-511^-P-eIF2-eIF2B complexes dataset

Movies were imported into Relion 5.0 (41) and motion corrected with Relion’s implementation of MotionCor using an EER fractionation of 40, 5x5 patches and dose-weighting. The motion corrected micrographs were then CTF corrected with CTFFIND4 (42). Particle picking was performed on the CTF corrected micrographs using Topaz (43) with a pre-trained agent. After applying a figure-of-merit cut-off of 1, 1184k particles were extracted with 400 pixels box size Fourier cropped to 100 pixels (bin4). Extracted particles were passed through 2D classification with 200 classes for 30 iterations using the old EM algorithm and fast subsets. 2D classes showing secondary structure features were selected (407k particles), re-extracted at unbinned pixel size and imported in cryoSPARC. Imported particles were passed through a second round of 2D classification with 50 classes. 326k particles were selected for non-uniform refinement, which resulted in an overall 3.04 Å resolution volume. A mask encompassing a single eIF2γ and eIF2Bγ subunits was created in ChimeraX (44). A symmetry expansion was then performed for a masked local refinement, followed by focused 3D classification using 6x classes. The highest populated class was used for local refinement (167k particles).

Fig. 3F is a composite map generated using ChimeraX by combining the original map and the local refinement map, applying the maximum value of each corresponding voxel across the two maps.

GSFSCs and local resolution estimations of reconstructions were calculated in cryoSPARC. Directional sphericity plots and sphericity values were calculated using a 3D-FSC server (https://3dfsc.salk.edu/) or publicly available code (https://github.com/nysbc/Anisotropy) (45).

### Model building

Initial models were generated with AlphaFold2 (46) for each subunit of eIF2 and eIF2B while R15B was predicted in the context of eIF2α and eIF2γ. Initial model docking was done in ChimeraX. All models were refined to their respective maps using Isolde (47). Further manual rebuilding and real-space refinement were conducted in COOT (48), with all-molecule self-restraints set to 5.0 for models with no sidechains. COOT refinements were performed using a German-McClure alpha parameter progressively increased from 0.01 to 1.

Final models were further refined in Refmac Servalcat (49) with its own restraint on. The Refmac-refined models were subsequently subjected to real-space refinement in Phenix (50). Restraint files for GTP were obtained from the COOT monomer library. Model validation was carried out using MolProbity (51).

Fig. 3G is a composite model generated by combining models reconstructed and refined from the original map and the local refinement map.

Fig. S5F is a composite model showing R15B residues 424-464 and 475-500. R15B residues 424-429 and 475-500 were modelled and refined from the cryo-EM density map of the R15B^411-511^-P-eIF2-eIF2B complex, as shown in Fig. 3H and fig. S5 D and E, and are shown as ribbon. R15B residues 430-464 were docked onto the surface of eIF2γ as predicted by AlphaFold2 and are shown with atomic surface coloured by Coulombic potential. eIF2α, eIF2β, eIF2γ and eIF2Bγ are modelled from the cryo-EM density map as in Fig. 3F and G. eIF2γ is shown with atomic surface coloured by Coulombic potential. Atomic surface Coulombic potential was estimated using ChimeraX.

Distance from R15B H1 to P-S52 in eIF2α in the R15B^411-511^-P-eIF2-eIF2B complex was measured with ChimeraX to be 107.2 Å.

Alignments in Fig. 3 J and K, fig. S3A and fig. S5C were generated using PyMOL (The PyMOL Molecular Graphics System, Version 3.1.3 Schrödinger, LLC) or ChimeraX (44). Fig. S5A was generated using ChimeraX to dock the structure of the R15B^411-511^-P-eIF2-eIF2B complex in the map of raised particles purified with R15B^414-713^.

Structural figure panels were generated using ChimeraX (44). 2D class cartoons and complex cartoons were generated using Adobe Illustrator.

### Cryo-EM 2D classification analyses

#### R15B^411-511, WT^-eIF2-eIF2B complexes

5697 movies were collected with 1.1 Å pixel size on Thermo Fisher Titan Krios 2 transmission electron microscope equipped with a Falcon4i electron detector camera. Movies were motion corrected using Relion’s implementation of MotionCor and then CTF-corrected with CTFFIND4 (42). Particle picking was done on the CTF-corrected micrographs using Topaz (43). Extraction at binning factor 4 (bin4) yielded 2544287 particles. From these, we used a randomized subset of 100k particles for 2D classification. Excluding low-resolution and junk classes, all 2D classes showed a signal for eIF2γ aligned against the body of eIF2B, the defining feature of the raised conformation.

#### R15B^411-511, H2A^-eIF2-eIF2B complexes

666 movies were collected with 1.58 Å pixel size on a Thermo Fisher Glacios transmission electron microscope equipped with an F3 electron detector camera. Movies were motion corrected using Relion’s implementation of MotionCor and then CTF-corrected with CTFFIND4 (42). Particle picking was done on the CTF-corrected micrographs using Topaz (43). After applying a figure-of-merit cut-off of 1, 122679 particles were extracted at bin4. A 1^st^ round of 2D classification was used to select particles showing secondary structure features. 41542 particles were selected and passed through a 2^nd^ round of 2D classification. From this, 11899 particles were in non-raised 2D classes, and 8515 particles were in raised 2D classes. The difference between these two classes was the presence (in raised classes) or the absence (in non-raised classes) of a visible signal for eIF2γ aligned against the body of eIF2B. The rest of the particles were low-resolution or junk classes.

#### R15B^414-713, KATA^-eIF2-eIF2B complexes

1159 movies were collected with 0.725 Å pixel size on a Thermo Fisher Krios transmission electron microscope equipped with a K3 electron detector camera. Movies were motion corrected using Relion’s implementation of MotionCor and then CTF-corrected with CTFFIND4 (42). Particle picking was done on the CTF-corrected micrographs using Topaz (43). After applying a figure-of-merit cut-off of 1, 36808 particles were extracted at bin4. A 1^st^ round of 2D classification was used to select particles showing secondary structure features. 18382 particles were selected. These particles underwent a 2^nd^ round of 2D classification. 10413 particles were in raised 2D classes. The rest of particles were low-resolution or junk classes.

#### R15B^414-713, KATA^-eIF2-eIF2B complexes after phosphatase treatment

3614 movies were collected with 0.725 Å pixel size on a Thermo Fisher Krios transmission electron microscope equipped with a K3 electron detector camera. Movies were motion corrected using Relion’s implementation of MotionCor and then CTF-corrected with CTFFIND4 (42). Particle picking was done on the CTF-corrected micrographs using Topaz (43). After applying a figure-of-merit cut-off of 1, 99365 particles were extracted at bin4. 2D classification resulted in 47211 particles classifying into catalytic 2D classes. The rest of the particles were low-resolution or junk classes.

### Protein identification by mass spectrometry

Gel samples were destained with 50% v/v acetonitrile and 50 mM ammonium bicarbonate, reduced with 10 mM DTT, and alkylated with 55 mM iodoacetamide. Next, bands were digested with 6 ng/μl trypsin (Promega, UK) overnight at 37 °C, and peptides extracted in 2% v/v formic acid 3% v/v acetonitrile and analysed, with a hybrid linear quadrupole ion trap mass spectrometer (Orbitrap QExactive, ThermoScientific, San Jose, USA). For R15B^411-511^ protein identification bands were digested with 6 ng/μl trypsin (Promega, UK) overnight at 37°C followed by 6 ng/μl chymotrypsin at 25 °C for 4 hours. Data-dependent analyses were carried out, using a resolution of 70,000 for the full MS spectrum, followed by ten MS/MS spectra in the linear ion trap. MS spectra were collected over a m/z range of 200–1800.MS/MS scans were collected using threshold energy of 35 for collision induced dissociation.

Tables S1, S2 and S4: Data were searched against the UniProt KB database using Mascot version 2.7 (Matrix Science), with a precursor tolerance of 5 ppm and a fragment ion mass tolerance of 0.8 Da. Two missed enzyme cleavages and variable modifications for oxidized methionine and carbamidomethyl cysteine were included. MS/MS data were validated using the Scaffold program (Proteome Software Inc). Data are exclusive unique peptide counts, which are the number of different amino acid sequences that are associated only with the protein identified.

Tables S5 to S8: Raw data files were processed in Proteome Discoverer v3.1 (Thermo Fisher Scientific). The raw files were submitted to a database search using Proteome Discoverer with SequestHF against the Uniprot reference proteome. Processing step consisted of a double iterative search using inferis rescoring on a first pass without any additional protein modifications and spectra with a confidence filter worse than "high" were researched with Sequest HT including additional common protein modifications (Deamidated N, Q; Oxidation M, gln to pyro-Glu Q; N-terminal acetylation; N-terminal acetylation and Methionine loss; Methionine loss. The spectra identification was performed with the following parameters: MS accuracy, 10 p.p.m.; MS/MS accuracy of 0.02 Da for spectra acquired in Orbitrap mass analyser; up to two missed cleavage sites allowed; carbamidomethylation of cysteine; and oxidation of methionine as variable modifications. Percolator node was used for false discovery rate estimation and only rank 1 peptide identifications of high confidence (FDR<1%) were accepted. Data are abundance values calculated using Proteome Discoverer v3.1 as the sum of the abundance of the three most abundant peptides for each specific protein. Abundance of each peptide is calculated as the integration of the signal of the peptide (intensity) over retention time.

### Immunoblots and antibodies

Immunopurified proteins from Expi293 cells were resolved in Bolt 4-12% Bis-Tris Plus gels (Invitrogen, #NW04125BOX) ran at 200 V for 30 min in 1X MES running buffer. Cell lysates and immunopurified proteins from HEK293T cells were resolved in Bolt 4-12% Bis-Tris Plus gels (Invitrogen, #NW04120BOX) ran at 120 V for 70 min in 1X MES running buffer. 2 μl of Protein Precision Plus Dual Colour Standards (#161-0374) was loaded on each gel. Separated proteins were transferred onto a nitrocellulose membrane (Bio-Rad, 1704159) using a Trans-Blot Turbo System (BioRad). To assess equal loading and quality of transfer, membranes were stained with Ponceau S solution (Sigma, P7170). Membranes were blocked for 30 min at RT in TBS with 0.025% Tween 20 (Sigma, P1379) (TBS-T) and 5% milk. Membranes were rinsed 3 times with TBS-T before overnight incubation at 4 °C with the relevant primary antibody diluted in 5% BSA in TBS-T. Membranes were washed 3 times with TBS-T before incubation at RT for 45 min with the appropriate horseradish peroxidase-conjugated secondary antibody (Promega, W4011, W4021), goat anti-mouse Alexa Fluor 680 (Invitrogen, #A32729, 1:5000) or goat anti-rabbit Alexa Fluor 790 (Invitrogen, #A27041, 1:10,000) diluted in TBS-T in 5% milk. Before imaging, membranes were washed 3 times with TBS-T and once with TBS. Amersham ECL Prime detection reagent kit (GE Healthcare Life Sciences, RPN2232) was used to detect chemiluminescence of horseradish peroxidase-conjugated secondary antibodies with ChemiDoc Touch imaging system (Bio-Rad). Membranes incubated with Alexa Fluor secondary antibodies were developed directly after washes with a ChemiDoc Touch imaging system (Bio-rad).

The following primary antibodies were used: eIF2α (Abcam, Ab26197, 1:1000; Cell Signaling, L57A5, 1:1000), P-eIF2α (Abcam, Ab32157, 1:1000), eIF2β (ProteinTech, 10227-1-AP, 1:1000), eIF2γ (Invitrogen, PA5-109680, 1:1000), eIF2Bα (ProteinTech, 18010-1-AP, 1:1000), eIF2Bβ (ProteinTech, 11034-1-AP 1:1000), eIF2Bγ (SinoBiological, 200321-T40, 1:1000), eIF2Bδ (AssaybioTech, C19274 1:1000), eIF2Bε (Abcam, Ab91565, 1:1000), Vinculin (Cell Signaling Technology, 4650S, 1:5000), FLAG (Sigma-Aldrich, F7425, 1:1000), PP1 (Santa Cruz, Sc-7482, 1:1000).

### Bioinformatics

The conservation scores of eIF2Bγ were computed using Consurf (52) and mapped to the sequence of eIF2Bγ using ChimeraX.

The sequence alignment of R15B homologs was adapted from (18).

### Quantification and statistical analysis

Western blots were quantified using ImageStudioLite or Image Lab Software (Bio-rad) and statistical analyses were performed using Prism 9.4.1/10 (GraphPad Software, Inc). One-way ANOVA with Tukey’s multiple comparisons test was performed in Fig. 4C for all possible pairwise comparisons. Unpaired, two-tail t-tests were performed in Fig. 4 E and G. Paired, two-tail t-tests were performed in Fig. 5 C, I and N. One-way ANOVA with Dunnett’s multiple comparisons test were performed in Fig. 5 L and M for comparing mutants to control. Data are presented as means ± SD for n≤ 3 and ± SEM for n ≥ 4.

## Supplementary Material

Tables S1-S2, S4-S8

Supplementary Materials

## Data Availability

Structure coordinates and cryo-EM maps have been deposited in the Protein Data Bank (PDB) and in the Electron Microscopy Data Bank (EMDB) under the accession codes: 9HVD, EMD-52432 (Native human P-eIF2-eIF2B complex), 9HVE, EMD-52433 (Native human eIF2-eIF2B complex), 9HVF, EMD-52434 (Native human PPP1R15B-P-eIF2-eIF2B complex). All other data is available in the main text or the supplementary materials. Materials used in this study will be made available upon request.
