# Supplementary material for: Termination of the integrated stress response
Source: Science. Author manuscript; Available in PMC 2025 Dec 25. (PMC7618491; doi:10.1126/science.adw5137)
Supplement: Supplementary Materials [file EMS210837-supplement-Supplementary_Materials.pdf]

Science
AAAS

Supplementary Materials for

Termination of the integrated stress response

Authors: Claudia De Miguel, Sigurdur R. Thorkelsson, Agnieszka Fatalska, George Hodgson, Chao Wang, Anne Bertolotti

Corresponding author: Anne Bertolotti

The PDF file includes:

Figs. S1 to S9

Table S3

Other Supplementary Materials for this manuscript include the following:

Tables S1, S2, S4 to S8

Fig. S1. Overexpressed R15B, R15A and native R15B immunoprecipitate eIF2B and eIF2.

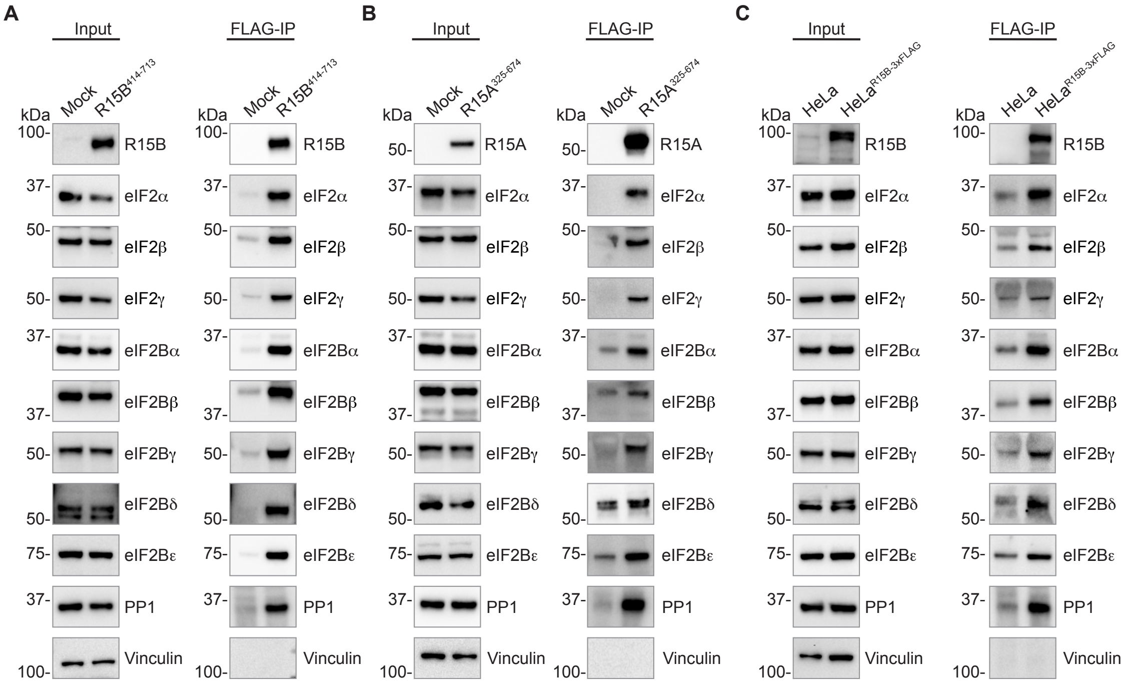

(**A**) Inputs and eluates of FLAG-R15B$^{414\text{-}713}$ immunoprecipitated complexes from HEK293T cells analysed on 4%–12% Bis-Tris Plus gels and revealed by immunoblotting with indicated antibodies. Representative results of n>3. (**B**) Same as in (A) with FLAG- R15A$^{325\text{-}674}$. (**C**) Same as in (A) and (B) with CRISPR FLAG-tagged endogenous R15B. Representative results of n>3.

Fig. S2. Cryo-EM data processing of the eIF2-eIF2B complex bound to R15B$^{414\text{-}713}$.

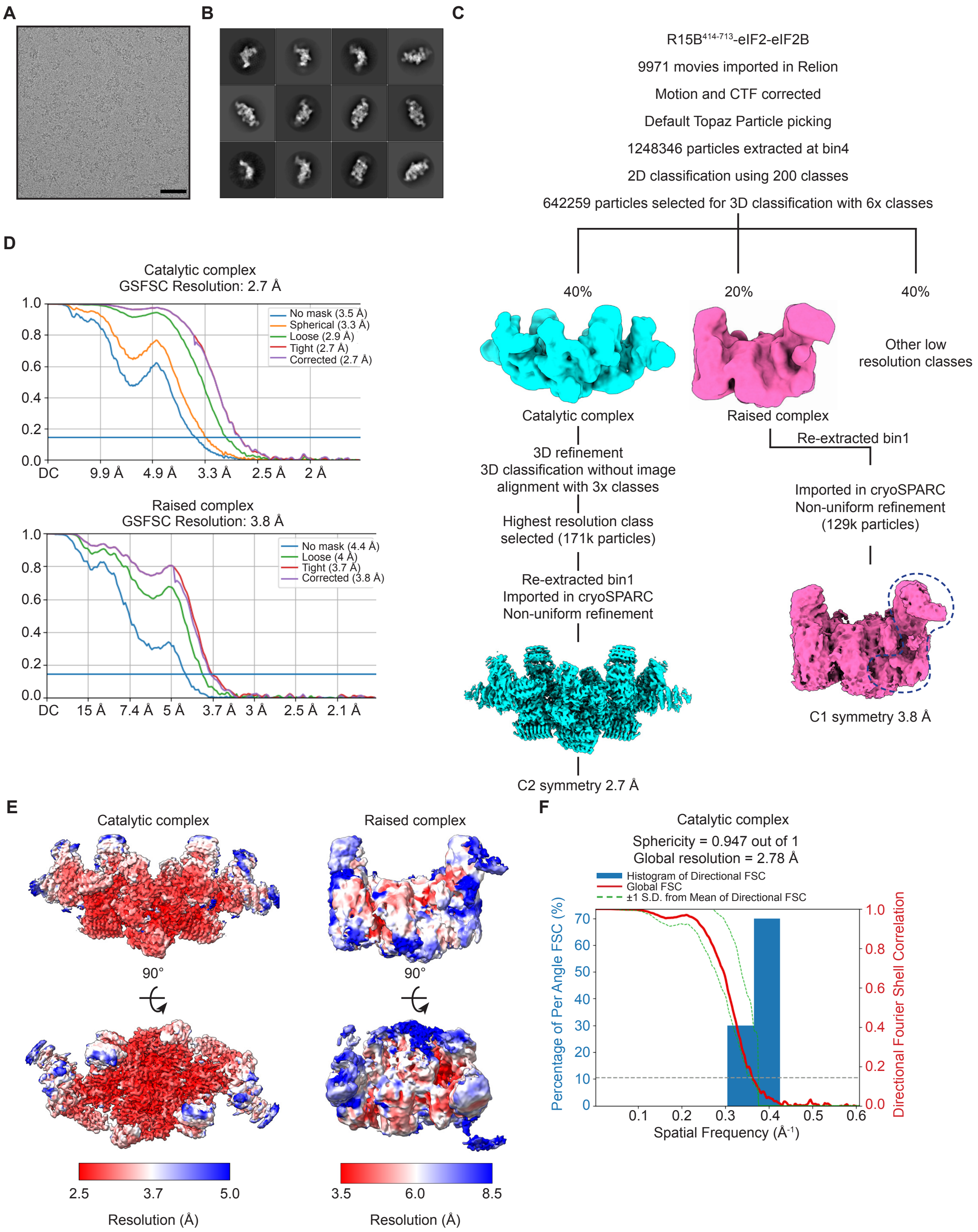

(**A**) Representative motion corrected micrograph. Scale bar = 50 nm. (**B**) Representative 2D class averages of R15B$^{414\text{-}713}$-eIF2-eIF2B particles. (**C**) Workflow of cryo-EM image processing as described in methods and table S3. A dashed outline highlights an eIF2 trimer in the raised eIF2B

complex. (**D**) Gold-standard Fourier Shell Correlation (GSFSC) plots for corrected (violet), unmasked (blue) and spherical (orange), tight (red) and loose (green) masked maps. (**E**) Local resolution maps of catalytic and raised complexes from two different views. (**F**) Directional sphericity plot and sphericity value (*45*) for the catalytic complex map.

Fig. S3. Structural features of R15B$^{414\text{-}713}$-eIF2-eIF2B complexes.

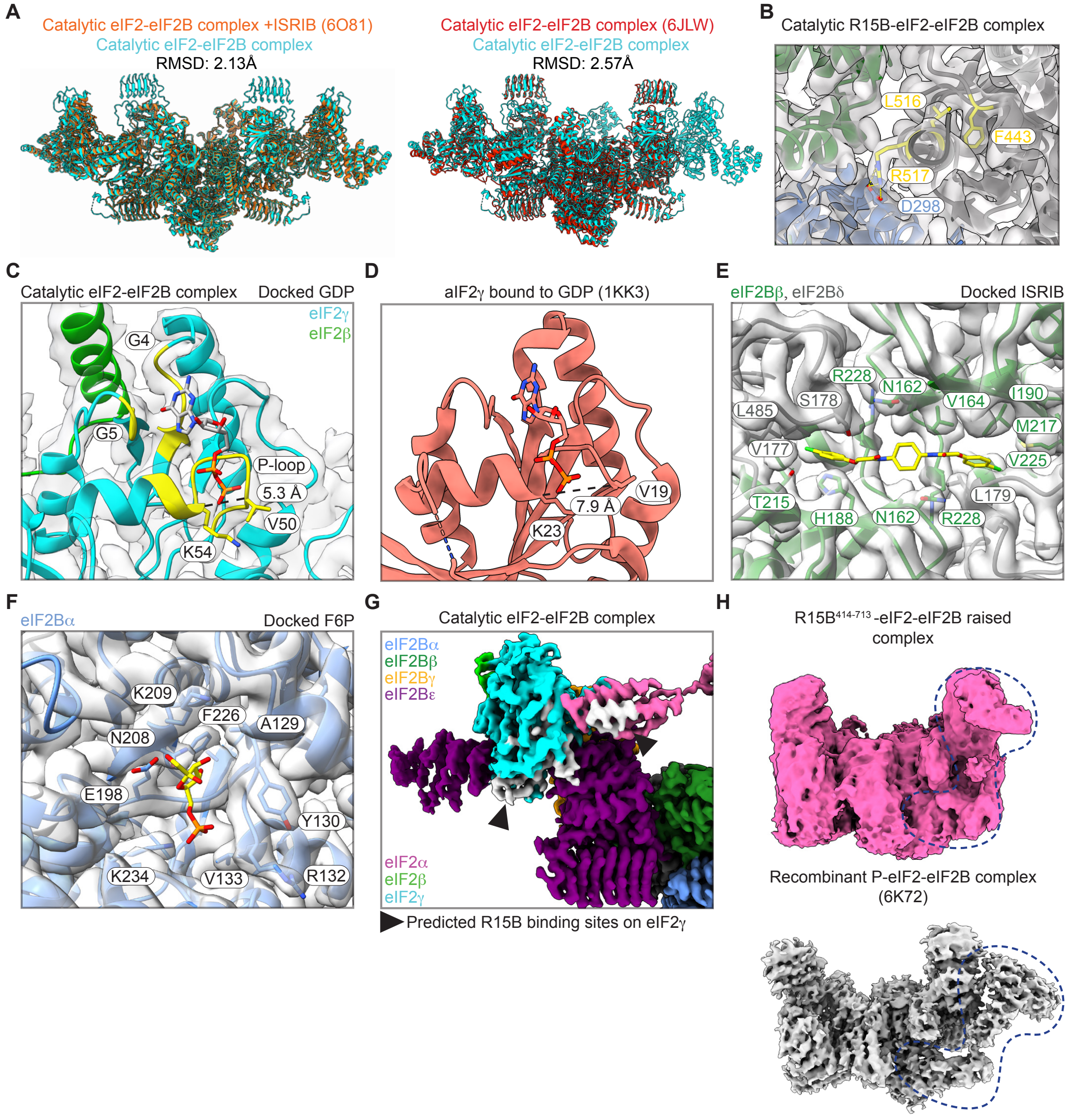

(**A**) The catalytic eIF2-eIF2B complex aligns with an RMSD of 2.13 Å to a catalytic eIF2-eIF2B complex bound to ISRIB (6O81) (left) and with an RMSD of 2.57 Å to a catalytic eIF2-eIF2B complex (6JLW) (right). (**B**) Close up view of the C-terminal α-helix of eIF2Bδ in the catalytic eIF2-eIF2B complex. R517 of eIF2Bδ forms a salt bridge with D298 of eIF2Bα, defining eIF2B in the active state. Key eIF2Bδ C-terminal α-helix residues highlighted in yellow. (**C**) Close-up of eIF2γ in the eIF2-eIF2B catalytic complex. There were no nucleotides bound to eIF2γ. For illustration purposes, a GDP molecule was docked based on a crystal structure of aIF2γ bound to

GDP (PDB: 1KK3). GDP binding was unfavourable in our structure because the diameter of the P-loop (5.3 Å, from V50 to K54) cannot accommodate the phosphate groups of the nucleotide. G domains of eIF2γ shown in yellow. (**D**) Structure of aIF2γ bound to GDP (PDB: 1KK3). The P-loop diameter (between V19 and K23) is 7.9 Å, which allows coordination of the phosphate groups of the GDP molecule. (**E**) ISRIB binding site (*24*) at the interface of eIF2Bβ and eIF2Bδ subunits was empty in the native eIF2-eIF2B complex. An ISRIB molecule (PDB: 6O81) was docked for visualisation of the binding site. (**F**) Sugar phosphate binding sites (*25*) on the two eIF2Bα subunits were empty in the native eIF2-eIF2B complex. Representative eIF2Bα subunit shown. F6P (PDB: 7KMF) was docked for visualisation of the binding site. (**G**) Close-up of the catalytic eIF2-eIF2B cryo-EM map with residues of eIF2 known to interact with R15B in a recombinant R15B-eIF2 complex (*18*) coloured in light grey and marked with arrowheads. R15B wasn't found in these predicted binding sites on eIF2. Instead, these sites were bound by eIF2Bε, inside eIF2γ or without additional binding densities in the catalytic complex. (**H**) Cryo-EM maps of a raised $R15B^{414\text{-}713}$-eIF2-eIF2B complex (top) and of a recombinant non-productive P-eIF2-eIF2B complex (PDB: 6K72) (bottom). Dashed lines indicate densities corresponding to eIF2.

Fig. S4. Cryo-EM data processing of the R15B$^{411\text{-}511}$-P-eIF2-eIF2B complex.

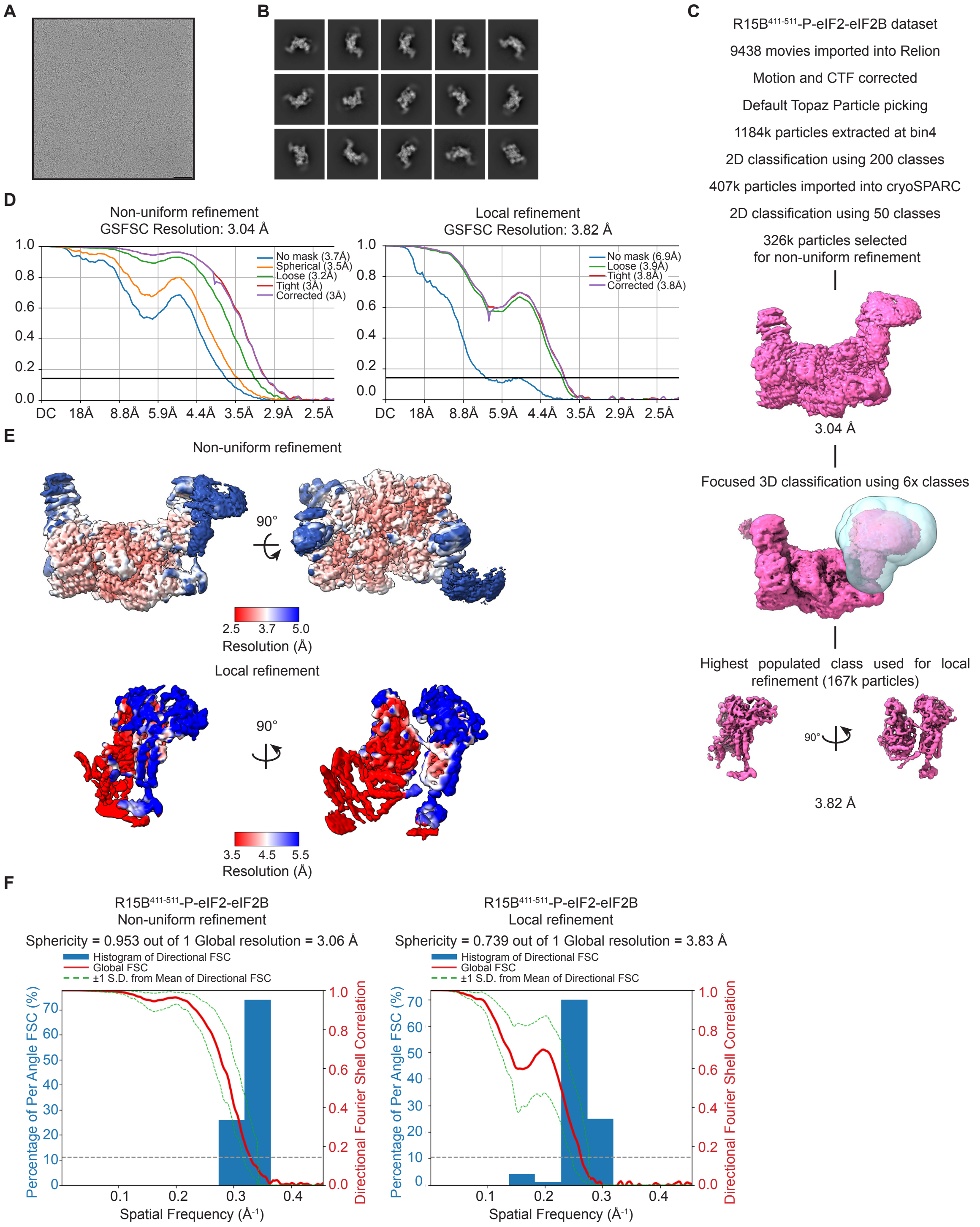

(**A**) Representative motion corrected micrograph. Scale bar = 50 nm. (**B**) Representative 2D class averages used for non-uniform refinement. (**C**) Workflow of cryo-EM image processing as described in methods and table S3. (**D**) Gold-standard Fourier Shell Correlation (GSFSC) plots of

corrected (violet), unmasked (blue) and spherical (orange), tight (red) and loose (green) masked maps of non-uniform refinement reconstruction (top) and local refinement reconstruction (bottom). (**E**) Local resolution maps of non-uniform refinement (top) and local refinement (bottom) reconstructions, from two different views. (**F**) Directional sphericity plots and sphericity values (*45*) for non-uniform refinement (left) and local refinement (right) maps.

Fig. S5. Structural features of the R15B$^{411\text{-}511}$-P-eIF2-eIF2B complex.

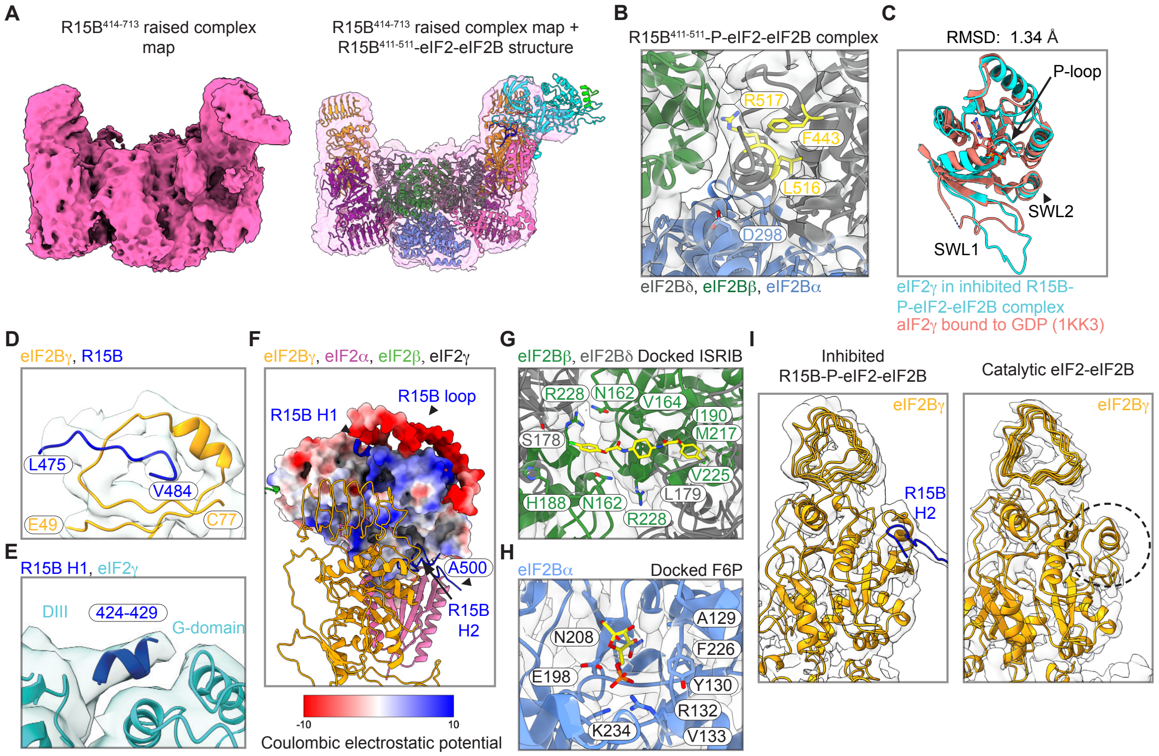

(**A**) Cryo-EM map of the raised R15B$^{414\text{-}713}$-eIF2-eIF2B complex from Fig. 2A (left). The structure of R15B$^{411\text{-}511}$-P-eIF2-eIF2B fits well into the raised particles purified with R15B$^{414\text{-}713}$ (right). (**B**) Close up view of the C-terminal α-helix of eIF2Bδ, located at the interface between eIF2Bδ, eIF2Bβ and eIF2Bα subunits in the raised R15B$^{411\text{-}511}$-P-eIF2-eIF2B complex. R517 in the C-terminal α-helix of eIF2Bδ extends away from D298 of eIF2Bα, defining eIF2B in the I state. Key eIF2Bδ C-terminal α-helix residues highlighted in yellow. (**C**) The G domain of eIF2γ in the R15B$^{411\text{-}511}$-P-eIF2-eIF2B (blue) inhibited complex aligned well (RMSD: 1.34 Å) with the G-domain of archaeal IF2γ bound to GDP (salmon) (1KK3). (**D**) R15B H2 (blue) binds the N-terminal domain of eIF2Bγ (yellow). (**E**) AlphaFold2 modelling of R15B$^{414\text{-}500}$ (predicted in the context of eIF2) docks R15B H1 (424-429, shown) to a density in the cryo-EM map of R15B$^{411\text{-}511}$-P-eIF2-eIF2B that extends across domains G and III of eIF2γ. (**F**) Composite structural model

of the R15B$^{411\text{-}511}$-P-eIF2-eIF2B complex and AlphaFold2 model of R15B residues 430-464 docked on eIF2γ. Atomic surface of eIF2γ and R15B$^{430\text{-}464}$ coloured by Coulombic potential. (**G**) ISRIB binding site (*24*) at the interface of eIF2Bβ and eIF2Bδ subunits was empty in the R15B$^{411\text{-}511}$-P-eIF2-eIF2B complex. An ISRIB molecule (PDB: 6O81) was docked for visualisation of the binding site. (**H**) Sugar phosphate binding sites (*25*) on the two eIF2Bα subunits were empty in the R15B$^{411\text{-}511}$-P-eIF2-eIF2B complex. Representative eIF2Bα subunit shown. F6P (PDB: 7KMF) was docked for visualisation of the binding site. (**I**) R15B binding site on eIF2Bγ in the inhibited complex (left). Same view of eIF2Bγ in the catalytic complex (right). R15B density is missing in the catalytic complex (dashed circle).

Fig. S6. Mutations of R15B destabilise the R15B-eIF2-eIF2B complex.

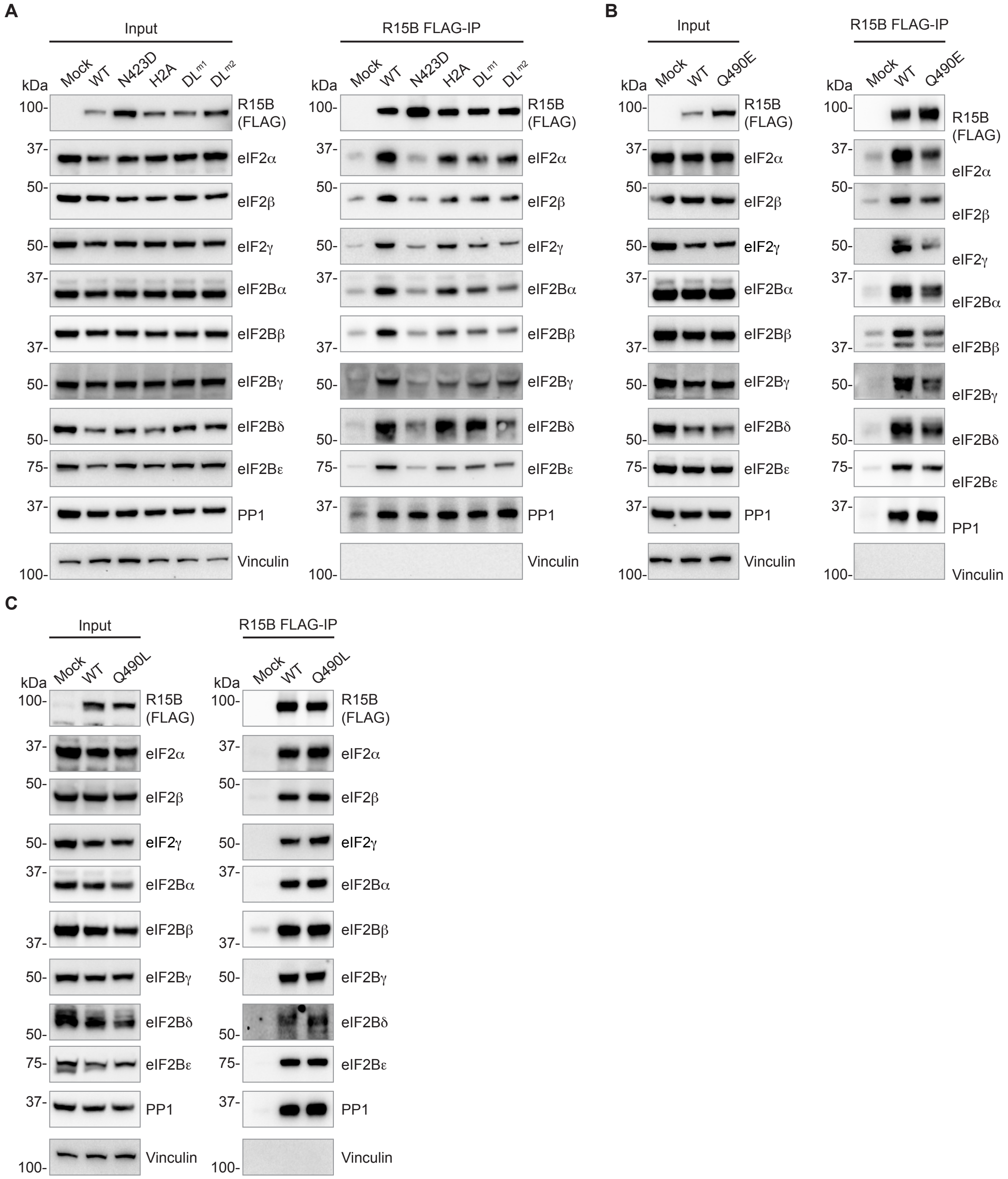

(**A** to **C**) Related to Fig. 4. Inputs and eluates of FLAG-R15B WT and mutants immunoprecipitated from HEK293T cells, analysed on 4%–12% Bis-Tris Plus gels and revealed by immunoblotting with indicated antibodies. Representative results of n>3.

Fig. S7. Purification of R15B$^{411\text{-}511}$-P-eIF2-eIF2B wild-type and H2A complexes.

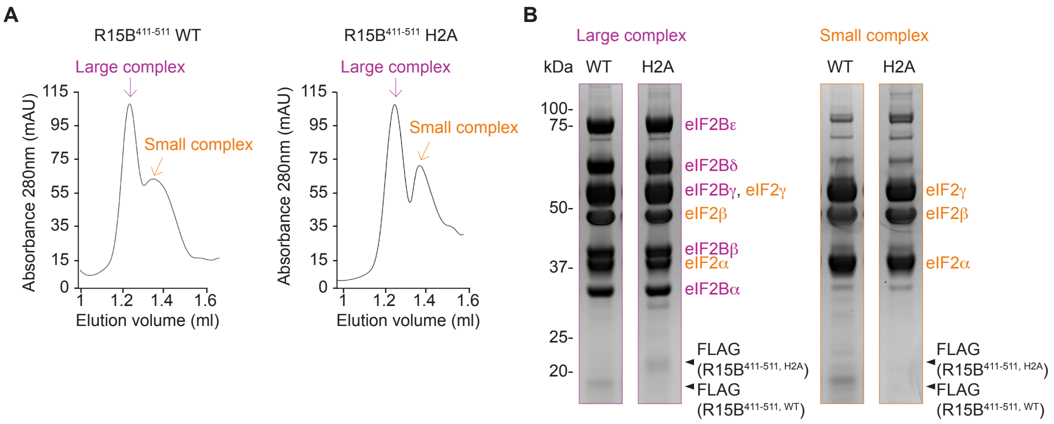

(**A**) SEC profiles of eluates immunopurified with FLAG-R15B$^{411\text{-}511}$ wild-type or H2A mutant from Expi293 cells. Peaks corresponding to large and small complexes indicated. (**B**) Coomassie stained 4%–12% Bis-Tris Plus gels of large and small complex peak fractions from SEC profiles shown in (A) (tables S7 and S8).

Fig. S8. C-terminal deletions of R15B progressively decrease its ability to enable dephosphorylation of P-eIF2 on eIF2B.

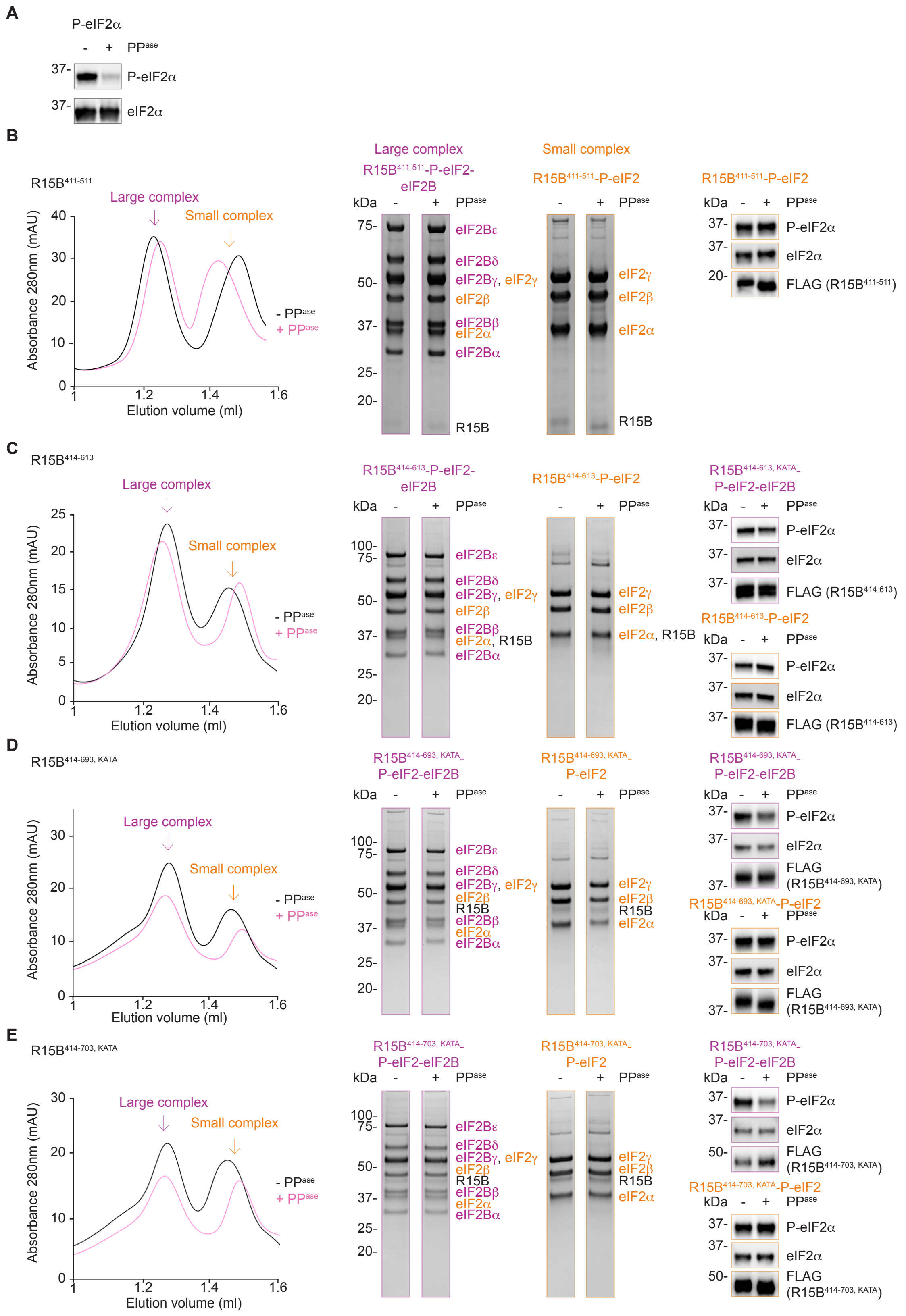

(**A**) Immunoblots with indicated antibodies after recombinant P-eIF2α was treated with or without alkaline phosphatase (AP) for 45 minutes at 30 °C degrees. (**B**) SEC profiles of FLAG-R15B$^{411-511}$ immunoprecipitated complexes after elution in the absence (black) or presence (pink) of AP for 45 minutes at 30 °C degrees. Representative SEC profiles of n=4 (left). Coomassie stained 4%–12% Bis-Tris Plus gels of the large and small complex from SEC (centre). Immunoblots with indicated antibodies of the small complex with or without phosphatase treatment (n=4) (right). (**C**) Same as in (B) with FLAG-R15B$^{414-613}$. Immunoblots with indicated antibodies of the large and small complex with or without phosphatase treatment (n=4) (right) (**D**) Same as in (C) with FLAG-R15B$^{414-693, KATA}$ (**E**) Same as in (C) and (D) with FLAG-R15B$^{414-703, KATA}$.

Fig. S9. Cartoons depicting the mechanism by which R15B relieves eIF2B from P-eIF2 inhibition to safeguard translation initiation.

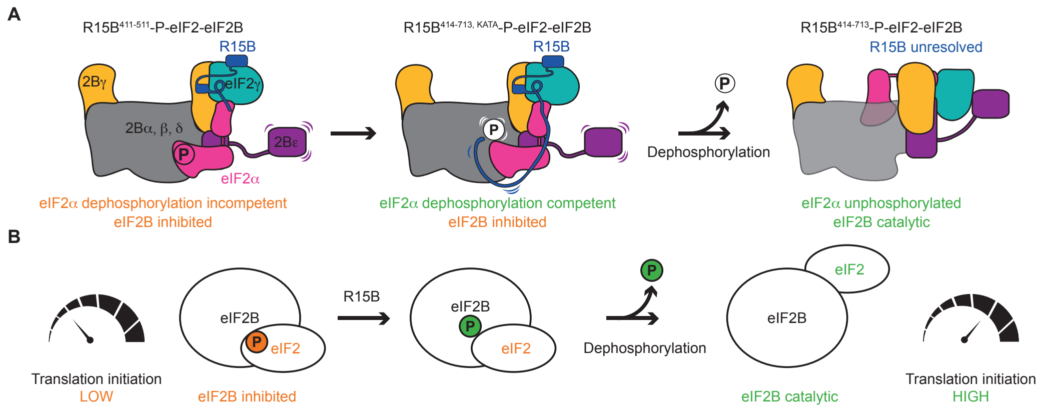

(**A**) From left to right. First panel: The structure of R15B$^{411-511}$ bound to the P-eIF2-eIF2B inhibited complex showed that R15B uses H1, H2 and the docking loop to tether eIF2Bγ to eIF2γ, causing eIF2γ and the adjacent C-terminal domain of eIF2α to align against the body of eIF2B in a raised conformation. The raised conformation holds eIF2B in an inhibited state as eIF2Bε can't access eIF2γ, which is kept in a GDP-bound conformation. In this complex, P-eIF2 is trapped in the inhibitory site of eIF2B with its P-S52 inaccessible to dephosphorylation. Second panel: R15B$^{414-713,\ KATA}$ enables dephosphorylation of P-eIF2 on eIF2B, most likely by inducing a conformational change to expose P-S52 on the P-eIF2-eIF2B complex. Third panel: Dephosphorylated eIF2 transfers from the inhibitory to a catalytic site of eIF2B, releasing eIF2B from P-eIF2 inhibition. In the resulting catalytic state, eIF2γ is sandwiched between eIF2Bε core and GEF domains. For simplicity, only one eIF2 is shown on eIF2B. Subunits coloured as in Fig. 3M. (**B**) Binding of P-eIF2 to eIF2B controls translation initiation rates. R15B rescues eIF2B from P-eIF2 by binding to the P-eIF2-eIF2B complex and enabling dephosphorylation of P-eIF2 on eIF2B. Dephosphorylation releases eIF2 from the inhibitory site of eIF2B, allowing its transfer to a catalytic site of eIF2B. Thus, R15B enables dephosphorylation of P-eIF2 on eIF2B to convert the inhibitor P-eIF2 into a substrate.

Table S3. Cryo-EM data collection, processing and model refinement.

**Data collection and processing**	Native human eIF2-eIF2B (9HVE)	Native human P-eIF2-eIF2B (9HVD)	Native human PPP1R15B-P-eIF2-eIF2B (9HVF)
Microscope	Titan Krios	Titan Krios	
Voltage (kV)	300	300	
Camera	Gatan K3	Falcon4i	
Magnification	105000x	75000x	
Pixel size at detector (Å)	0.824	1.10	
Electron exposure (e^-/Å^2)	50	50	
Number of frames	50	50	
Defocus range (mm)	-1 to -3	-0.8 to 2.4	
Automation software	EPU	EPU	
Energy filter slit width (eV)	20	/	
Movies collected (no.)	9971	9438	
Final particle images (no.)	171,151	326,401	167,091
Point-group	C2	C1	
Map resolution Å (unmasked/masked)	3.5/2.7	3.7/3.0	6.9/3.8
FSC threshold	0.143	0.143	0.143
Resolution range (local, Å)	1.9-8	2.5-8	2.8-14
Map sharpening B-factor	-66.7	-117.3	-108.5
3DFSC Sphericity	0.947	0.953	0.739
**Model composition**			
Protein residues	5630	3997	1065
Ligands	2	0	0

**Refinement**			
Refinement package	phenix.real_space_refine	phenix.real_space_refine	phenix.real_space_refine
CC volume/mask	0.88/0.89	0.85/0.85	0.73/0.74
Model resolution (Å) (unmasked/masked)	3.1/2.8	3.4/3.3	4.5/4.4
FSC threshold	0.5	0.5	0.5
B factors (Å^2)			
Protein residues (min/max/mean)	10.89/336.92/114.92	67.85/499.37/185.13	72.61/301.65/156.07
Ligand	108.57/184.26/136.71	/	/
R.m.s. deviations from ideal values			
Bond lengths (Å) (# > 4σ)	0.003	0.003	0.003
Bond angles (º) (# > 4σ)	0.584	0.601	0.759
**Validation**			
MolProbity score	1.82	1.62	2.51
CaBLAM outliers (%)	2.48	3.07	7.43
Clashscore	6.29	3.79	11.89
Poor rotamers (%)	2.12	1.18	3.05
Ramachandran plot			
Favoured (%)	96.54	94.10	89.91
Allowed (%)	3.35	5.57	9.23
Outliers (%)	0.11	0.33	0.86

Supplementary materials

Table S1 (separate file). Corresponding to Fig. 1B. Mass spectrometry analysis of proteins from visible bands excised from a Coomassie gel of the eluate immunopurified with FLAG-R15B$^{414-713}$.

Table S2 (separate file). Corresponding to Fig. 1D. Mass spectrometry analysis of proteins from visible bands excised from a Coomassie gel of large and small complexes purified with FLAG-R15B$^{414-713}$.

Table S4 (separate file). Corresponding to Fig. 3D. Mass spectrometry analysis of proteins from visible bands excised from a Coomassie gel of the large complex immunopurified with FLAG-R15B$^{411-511}$.

Table S5 (separate file). Corresponding to Fig. 3D. Mass spectrometry analysis of proteins from visible bands excised from a Coomassie gel of the large complex immunopurified with FLAG-R15B$^{411-511}$. Proteins were digested in the presence of both trypsin and chymotrypsin.

Table S6 (separate file). Corresponding to Fig. 3D. Mass spectrometry analysis of proteins from visible bands excised from a Coomassie gel of the small complex immunopurified with FLAG-R15B$^{411-511}$.

Table S7 (separate file). Corresponding to fig. S7B. Mass spectrometry analysis of proteins from visible bands excised from a Coomassie gel of the large complex immunopurified with FLAG-R15B$^{411-511, H2A}$.

Table S8 (separate file). Corresponding to fig. S7B. Mass spectrometry analysis of proteins from visible bands excised from a Coomassie gel of the small complex immunopurified with FLAG-R15B$^{411-511, H2A}$.
